# Transformer-based hand gesture recognition from instantaneous to fused neural decomposition of high-density EMG signals

**DOI:** 10.1038/s41598-023-36490-w

**Published:** 2023-07-07

**Authors:** Mansooreh Montazerin, Elahe Rahimian, Farnoosh Naderkhani, S. Farokh Atashzar, Svetlana Yanushkevich, Arash Mohammadi

**Affiliations:** 1 Department of Electrical and Computer Engineering, Concordia University, Montreal, QC Canada; 2 Concordia Institute for Information Systems Engineering, Concordia University, Montreal, QC Canada; 3 Departments of Electrical and Computer Engineering, Mechanical and Aerospace Engineering, New York University (NYU), New York, 10003 NY USA; 4 NYU Center for Urban Science and Progress (CUSP), NYU WIRELESS, New York University (NYU), New York, 10003 NY USA; 5 Biometric Technologies Laboratory, Department of Electrical and Software Engineering, Schulich School of Engineering, University of Calgary, Calgary, AB Canada

**Keywords:** Computational models, Data processing

## Abstract

Designing efficient and labor-saving prosthetic hands requires powerful hand gesture recognition algorithms that can achieve high accuracy with limited complexity and latency. In this context, the paper proposes a Compact Transformer-based Hand Gesture Recognition framework referred to as $\text {CT-HGR}$, which employs a vision transformer network to conduct hand gesture recognition using high-density surface EMG (HD-sEMG) signals. Taking advantage of the attention mechanism, which is incorporated into the transformer architectures, our proposed $\text {CT-HGR}$ framework overcomes major constraints associated with most of the existing deep learning models such as model complexity; requiring feature engineering; inability to consider both temporal and spatial information of HD-sEMG signals, and requiring a large number of training samples. The attention mechanism in the proposed model identifies similarities among different data segments with a greater capacity for parallel computations and addresses the memory limitation problems while dealing with inputs of large sequence lengths. $\text {CT-HGR}$ can be trained from scratch without any need for transfer learning and can simultaneously extract both temporal and spatial features of HD-sEMG data. Additionally, the $\text {CT-HGR}$ framework can perform instantaneous recognition using sEMG image spatially composed from HD-sEMG signals. A variant of the $\text {CT-HGR}$ is also designed to incorporate microscopic neural drive information in the form of Motor Unit Spike Trains (MUSTs) extracted from HD-sEMG signals using Blind Source Separation (BSS). This variant is combined with its baseline version via a hybrid architecture to evaluate potentials of fusing macroscopic and microscopic neural drive information. The utilized HD-sEMG dataset involves 128 electrodes that collect the signals related to 65 isometric hand gestures of 20 subjects. The proposed $\text {CT-HGR}$ framework is applied to 31.25, 62.5, 125, 250 ms window sizes of the above-mentioned dataset utilizing 32, 64, 128 electrode channels. Our results are obtained via 5-fold cross-validation by first applying the proposed framework on the dataset of each subject separately and then, averaging the accuracies among all the subjects. The average accuracy over all the participants using 32 electrodes and a window size of 31.25 ms is 86.23%, which gradually increases till reaching 91.98% for 128 electrodes and a window size of 250 ms. The $\text {CT-HGR}$ achieves accuracy of 89.13% for instantaneous recognition based on a single frame of HD-sEMG image. The proposed model is statistically compared with a 3D Convolutional Neural Network (CNN) and two different variants of Support Vector Machine (SVM) and Linear Discriminant Analysis (LDA) models. The accuracy results for each of the above-mentioned models are paired with their precision, recall, F1 score, required memory, and train/test times. The results corroborate effectiveness of the proposed $\text {CT-HGR}$ framework compared to its counterparts.

## Introduction

Hand gesture recognition using surface Electromyogram (sEMG) signals can be considered as one of the most important technologies in making efficient Human Machine Interface (HMI) systems. Hand gesture recognition-based HMI systems are applicable to a wide range of applications including prosthetics, neurorobotics, exoskeletons, and in Mixed (Augmented/Virtual) Reality settings, some of which targeting able-bodied individuals. In particular, sEMG-based hand gesture has been a topic of growing interest for development of assistive systems to help individuals with amputated limbs. Generally speaking, myoelectric prosthetic devices work by classifying existing patterns of the collected sEMG signals and synthesizing the intended gestures^1^. While conventional myoelectric control systems, e.g., on/off control or direct-proportional, have potential advantages, challenges such as limited Degree of Freedom (DoF) due to crosstalk have resulted in the emergence of data-driven solutions. More specifically, to improve efficiency, intuitiveness, and the control performance of hand prosthetic systems, several Artificial Intelligence (AI) algorithms ranging from conventional Machine Learning (ML) models to highly complicated Deep Neural Network (DNN) architectures have been designed for sEMG-based hand gesture recognition in myoelectric prosthetic devices^2–5^. The ML-based models encompass traditional approaches such as Support Vector Machines (SVMs), Linear Discriminant Analysis (LDA), and *k*-Nearest Neighbors (kNNs)^6–9^, and DNN-based models consist of frameworks such as Convolutional Neural Networks (CNNs), Recurrent Neural Networks (RNNs), and Transformer-based architectures^10–15^.

sEMG signals represent the electrical activities of the muscles and are recorded by a set of non-invasive electrodes that are placed on the muscle tissue^16,17^. Broadly speaking, there are two types of sEMG acquisition systems, called sparse and high-density^18,19^. Both of these groups are obtained by placing electrodes on the surface of the muscle and recording the electrical activity of the muscle’s Motor Unit Action Potentials (MUAPs) in response to the neural signals. Unlike sparse sEMG acquisition that involves a limited number of electrodes to record muscle activities, High-density sEMG (HD-sEMG) signals are obtained through a two-dimensional (2D) grid of electrodes, which cover an area of the muscle tissue and a large number of associated motor units^20,21^. When comparing HD and sparse sEMG signals, it can be stated that more computational power is required for the signal processing and training stages when using HD-sEMG signals in contrast to the scenario where sparse sEMG signals are used. This point has also been observed in the prior works^1,22^, where it is stated that HD-sEMG-based interfaces result in more complex analog front-end and processing facilities leading to increase of the computation demand. It is, therefore, more difficult to design an ML/Deep Learning (DL)-based algorithm for hand gesture recognition from HD-sEMG signals. However, HD-sEMG signals are considered more potent than their sparse counterparts because of their ability to include both temporal and spatial information of muscle activities, which provides a high-resolution 3-dimensional signal (two dimensions in space and one in time)^23^. The HD-sEMG signal acquisition can evaluate functionality of the underlying neuromuscular system more precisely in terms of spatial resolution. Accordingly, developing an efficient DNN-based framework that can effectively learn from a comprehensive HD-sEMG dataset is of great importance in neuro-rehabilitation research and clinical trials^24^, which is the focus of this manuscript.

Conventional ML models, such as SVMs and LDAs, utilized for sEMG-based hand gesture recognition, typically work well when dealing with small datasets. These methods, however, depend on manual extraction of handcrafted (engineered) features, which limits their generalizability as human knowledge is needed to find the best set of features^25^. Increasing the number of utilized electrodes and the number of gestures entails extracting more features, therefore, the feature extraction process becomes significantly complex and time-consuming. This is because more trials and efforts are required to boost the discriminative power of the model. Dependence on engineered features is partially/fully relaxed by utilization of DNN-based models. Among the most frequently used DNN architectures for the task of hand gesture recognition is the CNN-based frameworks. For example, Reference^12^ converts sEMG signals to 3D images and uses transfer learning to feed them to a popular CNN trained on a database of natural images. CNNs, however, are designed to concentrate on learning spatial features of the input signals and fail to extract temporal features of the sEMG data. To overcome this issue, researchers turned their attention to hybrid CNN-RNN frameworks that were designed to take both spatial and temporal information of the time-series sEMG datasets into account^26,27^. For instance, Hu *et al.*^26^ have applied attention mechanism on top of a hybrid CNN-LSTM (Long Short-Term Memory) model to perform hand gesture recognition based on sEMG signals with relatively large window sizes (i.e. 150 ms and 200 ms). They achieved classification accuracy of up to $87\%$ using the largest window size. In^27^, a dimensionality reduction method is proposed and assumed to enhance the classification accuracy when used with a hybrid CNN-LSTM architecture. In this framework^27^, the classification accuracy is $88.9\%$ on the same dataset as that of^26^ for the 250 ms window size. Nonetheless, as well as not allowing entire input parallelization, hybrid CNN-RNN frameworks are usually computationally demanding and reveal important limitations with respect to the memory usage and large training times. To alleviate the problem of lacking input parallelization in the aforementioned networks, References^15,28^ proposed transformer-based models for gesture recognition via sparse sEMG signals. For instance, in^28^ a Vision Transformer (ViT) network is stacked to CNNs for gesture classification using the frequency domain information (Fourier transform) of a set of sparse sEMG signals. In this study, first and different from these prior works, we target HD-sEMG signals. Second, by eliminating the complexity of simultaneously exploiting CNNs/RNNs or merging them with transformers, we aim to construct a compact and stand-alone framework with reduced computational overhead. When it comes to real-time HMI devices, we hypothesized that by introducing a compact DL-based model developed based on HD-sEMG signals that has the capacity to classify a large number of hand gestures with a small amount of memory and training time, we can put a step forward towards development of more dextrous control interfaces. On the one hand, while DL models are more complicated than conventional ML solutions, the latter requires operator interventions for feature engineering, which is a burdensome procedure. On the other hand, Gesture recognition based on sparse sEMG requires precisely locating the electrodes over the muscle to make sure that the same MUs are being recorded. Different from sparse sEMG, for the HD-sEMG acquisition, a little change in the position of the electrode grid still records the MU activities with no significant change in the characteristics of the signal.

In this study, a comprehensive evaluation of the proposed ViT-based framework for hand gesture classification on HD-sEMG dataset is carried out for the first time to the best of our knowledge. The ViT architecture takes advantage of the attention^29^ mechanism, which works by finding dependencies and similarities among different data portions. The attention mechanism in the ViT is integrated in a typical transformer model, making it a robust framework for hand gesture recognition without being combined with other DL algorithms. One of the differences between the ViT and a typical transformer is that the ViT is generally designed to be applied on 2D RGB images that have an additional dimension (the $3^{\textrm{rd}}$ dimension) as the color channel rather than 2D time-series signals. Considering the fact that HD-sEMG signals comprise of two dimensions in space and one in time (3 dimensions in total), they can be an appropriate input to a ViT. As mentioned in^30^, instantaneous training with HD-sEMG signals refers to training the network with a 2D image depicting MUAP activities under a grid of electrodes at a single time point. In this paper, we also show that there are reproducible patterns among instantaneous samples of a specific gesture which could also be a physiological representation of muscle activities in each time point. We demonstrate that the proposed framework can perform instantaneous hand gesture classification using sEMG image spatially composed from HD-sEMG. In other words, it can achieve acceptable accuracy when receiving, as an input, a single frame of the HD-sEMG image. The main contributions of the paper are briefly outlined below:

- To the best of our knowledge, the proposed Compact Transformer-based Hand Gesture Recognition framework ($\text {CT-HGR}$) is the first ViT-based architecture that is leveraged to classify hand gestures from HD-sEMG signals. It can efficiently classify a large number of hand gestures relying only on the attention mechanism. Furthermore, the $\text {CT-HGR}$ can be trained from scratch without the need for transfer learning or data augmentation.
- Achieving near baseline accuracy using instantaneous HD-sEMG data samples, which is significant as it paves the way for real-time learning from HD-sEMG signals.
- Introducing, for the first time to the best of our knowledge, the idea of integrating macroscopic and microscopic neural drive information through a hybrid DNN framework. The proposed variant of the $\text {CT-HGR}$ framework, is a hybrid model that simultaneously extracts a set of temporal and spatial features through its two independent ViT-based parallel architectures (the so called Macro and Micro paths). The Macro Path is the baseline $\text {CT-HGR}$ model, while the Micro path is fed with the peak-to-peak values of the extracted MUAPs of each source.

The rest of the paper is structured as follows: The utilized HD-sEMG dataset is introduced in Sub-Sect. "The HD-sEMG dataset". An explanation of the pre-processing procedures on the raw dataset is given in Sub-sect. "Data pre-processing" and our proposed framework is presented in Sub-sect. "The proposed CT-HGR". Our experiments and evaluations of implementing the proposed framework are discussed in Sect. "Results", a detailed discussion of the acquired results is generated in Sect. "Discussion" and finally, Sect. "Conclusion" concludes the paper.

## Materials and methods

### The HD-sEMG dataset

**Figure 1:**
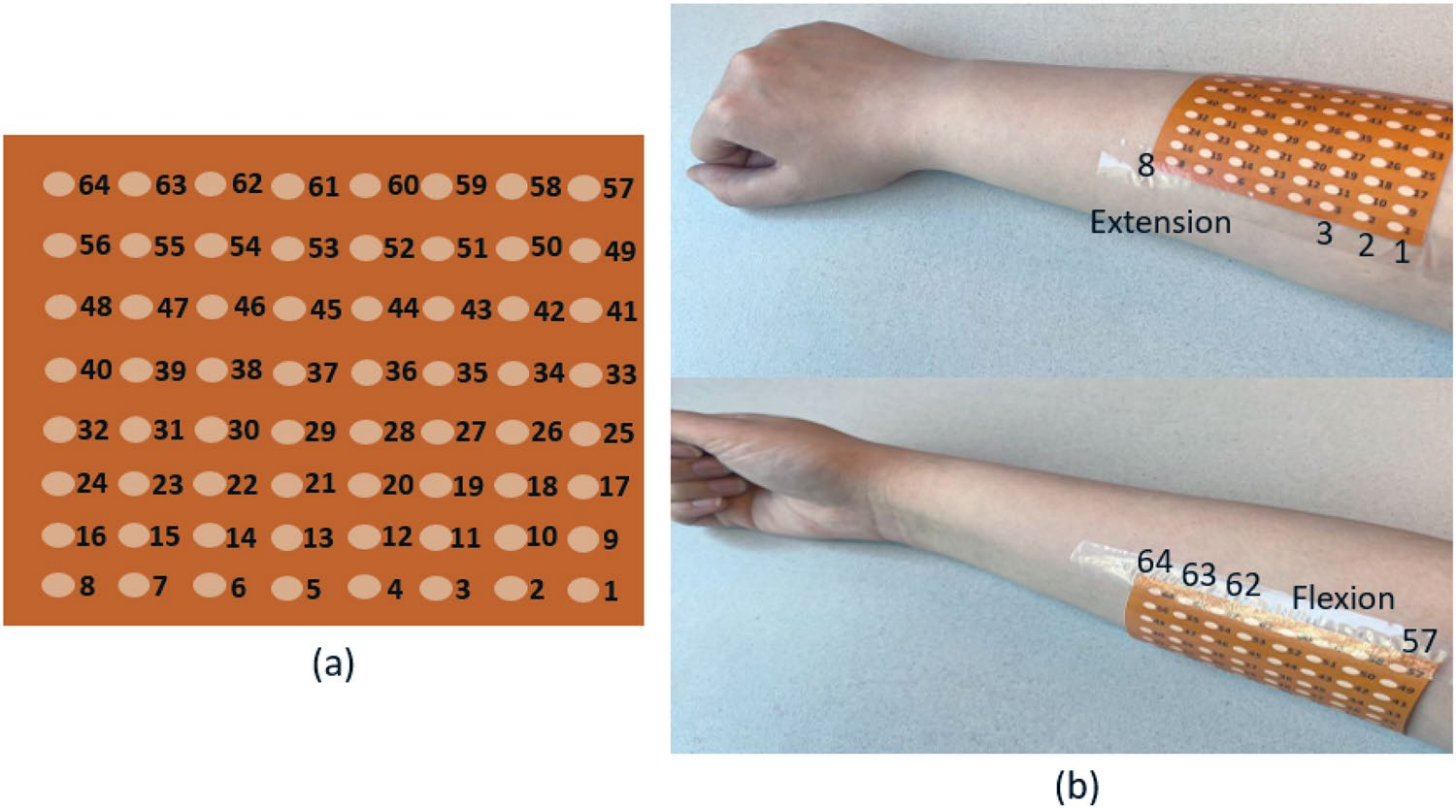
Representation of the HD-sEMG acquisition setup^31^: (**a**) The ($8\times 8$) HD-sEMG grid of electrodes. (**b**) The flexion and extension electrodes positioned on supinated and fully pronated forearm muscles.

**Figure 2:**
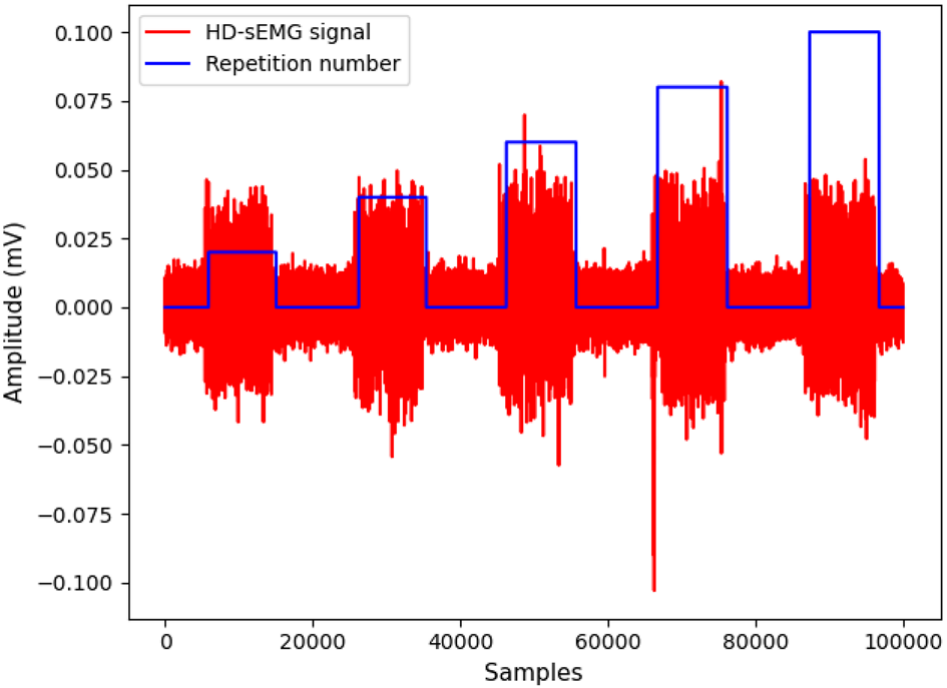
Illustrative example of the raw HD-sEMG dataset. The red plot is the sEMG signal for one single channel and one single movement and the blue plot shows the repetition number and the rest intervals for that movement.

The dataset^31^ used in this study is a recently released HD-sEMG dataset that contains two 64-electrode square grids ($8\times 8$) with an inter-electrode distance of 10 mm, which were placed on extensor and flexor muscles. The HD-sEMG acquisition setup is shown in Fig. 1. According to^31^, the two HD-sEMG electrode grids covered the dorsal and the volar muscles of the forearm, specifically full or partial parts of flexor digitorum profundus and flexor digitorum superficialis, which is for flexion of fingers D2-D5, extensor digitorum communis for extension of fingers D2-D5, flexor carpi radialis and flexor carpi ulnaris for wrist flexion, extensor carpi radialis longus and extensor carpi ulnaris for wrist extension, pronator teres, supinator, and flexor pollicis longus for thumb flexion, extensor pollicis longus for thumb extension and abductor pollicis longus. Data from 20 participants is provided through the dataset. One of the subjects is not included in the study from the beginning due to its incomplete information. The participants performed 65 hand gestures that are combinations of 16 basic single degree of freedom movements. One of the gestures is carried out twice, therefore, there are 66 movements in total. The subjects performed each gesture 5 times with 5 seconds rest in between. Fig. 2 illustrates how the raw dataset is organized. The red plot shows the acquired HD-sEMG signal for one single channel of one specific hand movement. The blue line shows the repetition number of that gesture and the rest intervals. The signals were recorded through a Quattrocento (OT Bioelettronica, Torino, Italy) bioelectrical amplifier system with 2, 048 Hz sampling frequency. Signals of the successive channels were subtracted from each other (i.e., the sEMG data is acquired in a bipolar fashion) to lower the amount of common-mode noise. The rational behind selection of this publicly available dataset is that it comprises of a large number of gestures and electrodes, which allows development of a generalizable framework by investigating different settings of the input data. Additionally, this dataset provides straightforward instructions on how to deploy the dataset for different evaluation purposes. However, since the paper^31^ on this dataset did not refer to the train and test sets as a basis for comparison, we performed a 5-fold cross-validation as there are 5 sessions in the dataset. In this way, one (out of 5) repetition is considered as the test set and the remaining are assigned to the train set. Each time, the test set is changed until all the repetitions have been tested. Finally, the accuracy of each fold together with the average accuracy across all the folds are reported.

### Data pre-processing

The raw HD-sEMG dataset is pre-processed following the common practice before being fed to the proposed $\text {CT-HGR}$ framework. More specifically, there is a consensus in the literature that pre-processing of sEMG signals should involve the following steps: (i) Band pass filtering; (ii) Rectification; (iii) Linear envelope computation, and (iv) Normalization. The utilized dataset is band-pass filtered with a hardware high-pass filter at 10 Hz and a low-pass filter at 900 Hz during recordings. All filter types are second order butterworth filters. Prior to the filtering step, full wave rectification is performed, i.e., absolute value of the signal is computed. The rectification step coupled with the low-pass filtering results in getting the shape or “envelope” of the sEMG signal. The envelope obtained by low-pass filtering is used to acquire active segment data^32,33^. The purpose of the low pass filtering is to attenuate higher frequencies present in the signal while keeping the DC and low frequency values. In this regard, a low-pass first-order butterworth filter at 1 Hz is applied separately to each of the 128 channels of the data. We would like to mention that the utilized low-pass filtering approach is common in the literature, e.g., References^34–37^ also applied a low-pass filter with cutoff frequency of 1 or 2 Hz and then windowed the signal. Shallower filters are widely recommended as they produce less signal distortions and spread them less in the time domain due to a shorter impulse response. Using the Fourier transform of the HD-sEMG signals^38,39^, we observed that the cut-off frequencies up to 10 Hz are reasonable, as such we have also tested the model’s performance for 5 and 10 Hz low-pass filters as shown in Table 1. It is worth nothing that low-pass filtering can be seen, more or less, to smoothing the data with a sliding averaging window. In this regard, theory predicts that a moving average filter will have a cutoff frequency equal to $f=\frac{0.443}{T_{w}}$ (e.g., a moving average filter with 1 Hz cutoff frequency corresponds to a 443 ms window size). Having said that, Butterworth filter in the time domain has an infinite impulse response with positive and negative lobes in contrast to the moving average filter, which is a finite positive window with constant values in time. Intuitively speaking, the positive and negative lobes of the butterworth filter neutralize the effect of averaging over time instants. In final pre-processing phase, the filtered signals are normalized by the $\mu$-law normalization algorithm, which reduces significant changes in the dynamic range of the signals acquired from different electrodes. The $\mu$-law normalization is performed based on the following formulation

1 $$\begin{aligned} F(x_t) = \text {sign}(x_t)\frac{\ln {\big (1+ \mu |x_t|\big )}}{\ln {\big (1+ \mu \big )}}, \end{aligned}$$

where $x_t$ is the time-series sEMG signal for each electrode channel, and $\mu$ is the extent to which the signals are scaled down and is determined empirically. According to^2,40^, $\mu$-law normalization helps the network to learn gestures more effectively. Fig. 3 shows the effects of the $\mu$-law normalization. As can be seen from Fig. 3, original signals are closely spaced and their amplitudes change in a very small range (i.e. $\approx$ 0-0.02 V). They are, however, apparently separated after applying the $\mu$-law normalization, which results in the sEMG signals ranging from $\approx$ 15-50 V. Having separated values provide the network with better learning capabilities to discriminate between different gestures. Finally, the sEMG signals are segmented following the common approach in the literature^41–44^. More specifically, after removing the rest intervals from the dataset, the signals are segmented with a specific window size creating the main 3D input of the $\text {CT-HGR}$ with shape $W\times N_{ch}\times N_{cv}$, where *W* is the window size and $N_{ch}$ and $N_{cv}$ are the number of horizontal and vertical channels respectively. This completes our discussion on the pre-processing stage. In what follows, the proposed $\text {CT-HGR}$ framework is presented, which takes the pre-processed data samples as its input and returns the predicted gesture class.

**Table 1:** Comparison of classification accuracy and STD for each fold and their average for $W=64$, 128 electrode channels ($\text {CT-HGR}$-V1), and different cutoff frequencies for the low-pass filter. The accuracy and STD for each fold is averaged over 19 subjects.

# Channels	Window size (samples)	Cutoff freq(Hz)	Fold1(%)	Fold2 (%)	Fold3 (%)	Fold4 (%)	Fold5 (%)	Average (%)
128	64	1	82.14 (±3.26)	93.30 (±2.14)	93.75 (±2.08)	93.39 (±2.11)	90.07 (±2.55)	90.53 (±2.43)
		5	81.94 (±3.74)	92.74 (±2.46)	93.48 (±2.12)	93.33 (±2.10)	89.64 (±2.95)	90.23 (±2.67)
		10	80.40(±3.44)	91.42 (±2.38)	92.27 (±2.28)	91.98 (±2.28)	88.30 (±2.80)	88.87 (±2.64)

**Figure 3:**
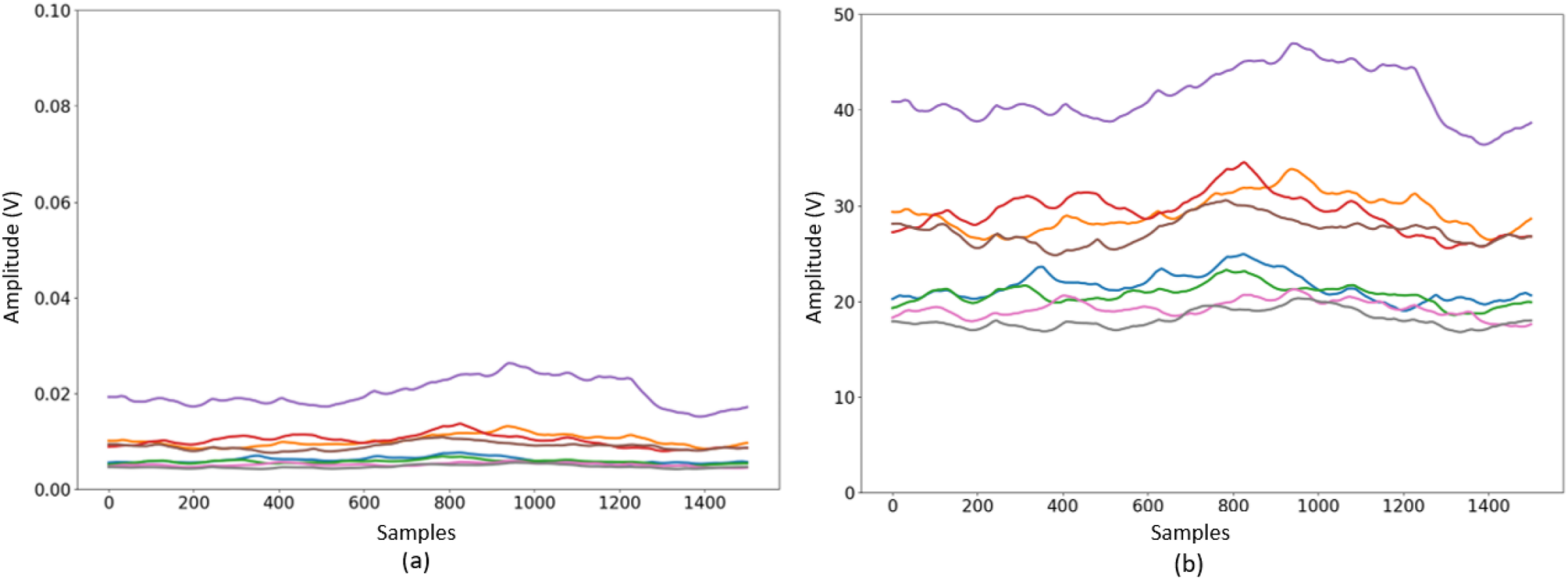
The impact of the $\mu$-law normalization on the sEMG signals: (**a**) Low-pass filtered sEMG signals of 8 different electrode channels of the extensor grid before normalization. (**b**) Low-pass filtered sEMG signals of 8 different electrode channels of the extensor grid after normalization.

### The proposed $\text {CT-HGR}$

In this section, description of our proposed $\text {CT-HGR}$ framework, its main building blocks, and its adoption for the task of hand gesture recognition are presented. The $\text {CT-HGR}$ is developed based on the ViT network in which the attention mechanism is utilized to understand the temporal and spatial connections among multiple data segments of the input. As stated previously, several studies have employed the attention mechanism together with hybrid CNN-RNN models to force the network to learn both spatial and temporal information of the signals^3,26^. However, in this paper, we demonstrate that attention mechanism can work independently of any other network and achieve high accuracy when trained from scratch with no data augmentation. We also show that the proposed framework can be trained even on small window sizes and more importantly on instantaneous data samples. It is worth noting that in the recent literature, there are some works^30,41^ that focused on small windows sizes achieving accuracies in the range of 89.3 - 91.81.

**Figure 4:**
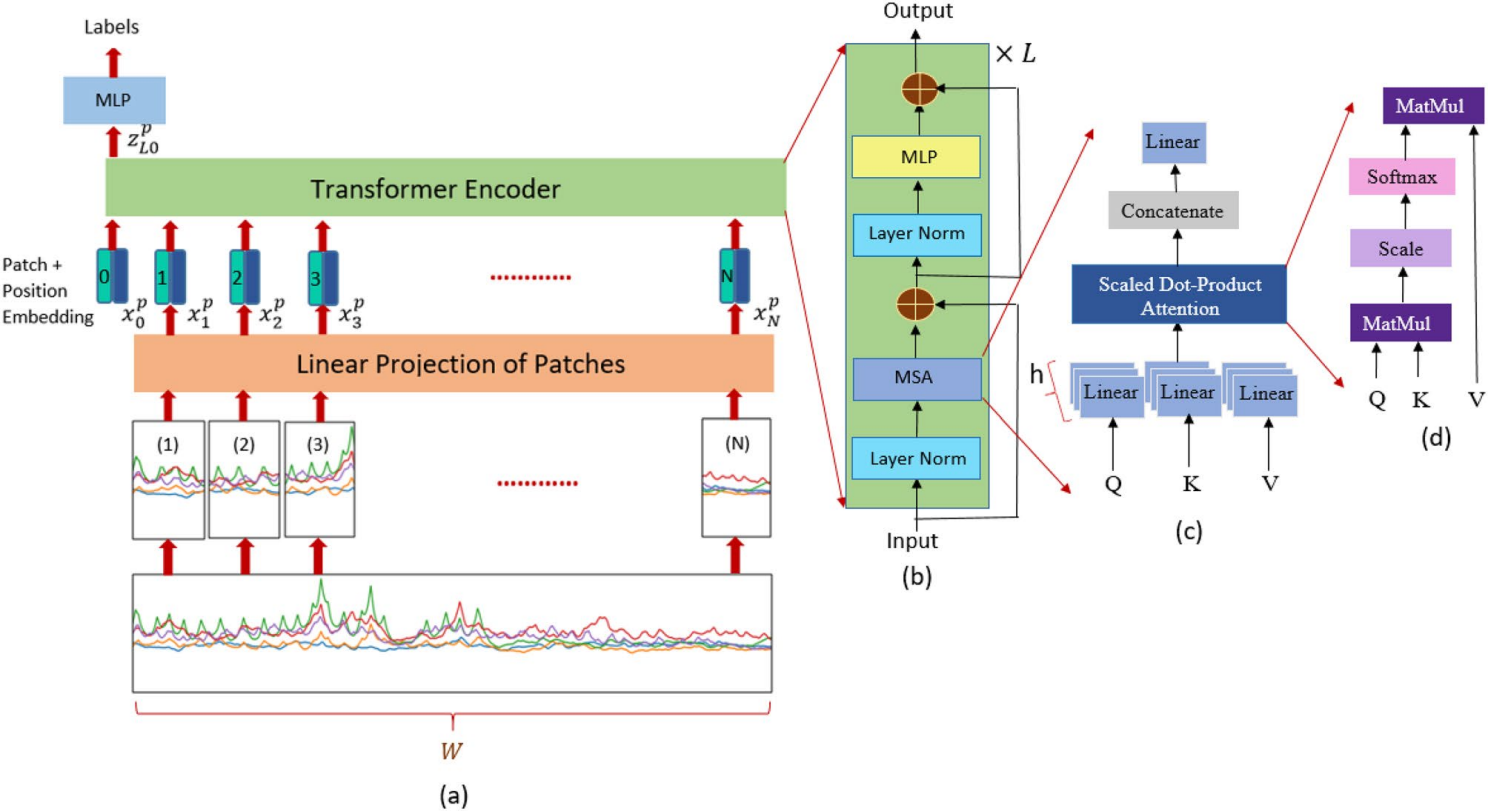
Overview of the $\text {CT-HGR}$ network. (**a**) The windowed HD-sEMG signal is fed to the $\text {CT-HGR}$ and split into smaller patches. The patches go through a linear projection layer which converts them from 3D to 2D data samples. A class token is added to the patches and the $N+1$ patches are input to a transformer encoder. Ultimately, the first output of the transformer corresponding to the class token is chosen for the multi-class classification part. (**b**) The transformer encoder which is the fundamental part of the ViT, responsible for processing the input patches with its main part called Multi-head Self Attention (MSA). (**c**) The Multi-head Self Attention (MSA) Structure. (**d**) The Scaled Dot-Product module in the MSA block.

An overall illustration of the $\text {CT-HGR}$ is indicated in Fig. 4. After completion of the pre-processing steps discussed in the previous section, we have 3D signals of shape $W\times N_{ch}\times N_{cv}$, where *W* is the window size and $N_{ch}$ and $N_{cv}$ are the number of horizontal and vertical channels respectively. As an intuitive approach for patching the input data with 32, 64 or 128 electrode channels, we considered window sizes that are powers of two (in samples), which allows to smoothly divide input into smaller patches^45^. Therefore, the utilized window sizes in our experiments are of 64, 128, 256, and 512 data points (31.25, 62.5, 125, and 250 ms respectively considering 2, 048 Hz sampling frequency of the dataset). Furthermore, we have assessed the effect of changing the number of electrode channels by using 32, 64 and 128 out of the whole 128 channels. Therefore, we set $N_{ch}$ to 4, 8, and 16 each time while $N_{cv}$ remains constant at 8. In what follows, the major blocks of the proposed $\text {CT-HGR}$ network, namely “Patch Embedding”, “Position Embedding”, “Transformer Encoder”, and the “Multilayer Perceptron (MLP)” blocks.

#### Patch embedding

In this block, the 3D signals are divided into *N* small patches either horizontally, vertically or both. Therefore, we have *N* patches of size $H\times V\times N_{cv}$ that are then linearly flattened to 2D signals of size $N\times HVN_{cv}$ where, *N* is equal to $WN_{ch}/HV$ and is the effective sequence length of the transformer’s input and terms *H* and *V* represent the horizontal and vertical patch sizes, respectively. Consequently, there are *N* patch vectors ${\varvec{x}}^{p}_{i}$, for ($1 \le i \le N$). Using a trainable linear projection layer, the ${\varvec{x}}^{p}_{i}$ vectors are embedded with the model’s dimension *d*. The linear projection is shown with matrix $\varvec{E}$, which is multiplied to each of the ${\varvec{x}}^{p}_{i}$ and yields *N* vectors of dimension *d*. Moreover, a class token named ${\varvec{x}}^{p}_{0}$ similar to what was previously used in the Bert framework^46^ is prepended to the aforementioned vectors to gather all the useful information learned during the training stage and is used in the final step when different hand gestures are classified. The final sequence length of the transformer after adding the class token is $N+1$.

#### Position embedding

Unlike RNNs that process their inputs sequentially, transformers apply the attention mechanism to all of the data segments in parallel, which deprives them of the capacity to intrinsically learn about the relative position of each patch of a single input. Because sEMG signals are time-series sequences of data points in which the location of each point matters for hand gesture classification tasks, we need to train the network to assign a specific position to each sample. Generally speaking, positional embedding is an additional piece of information that is injected into the network, helping it to identify how data points are ordered. There are different types of positional embeddings offered such as relative, 1D, 2D, and sinusoidal positional embeddings that may be learnable or non-learnable. In this context, we use a learnable 1D positional embedding vector that is added to each of the embedded ${\varvec{x}}^{p}_{i}$ vectors to maintain and learn the position of each patch during the training phase. The final output $z_0$ of the “Patch + Position Embedding” blocks is given by

2 $$\begin{aligned} z_0 = \left[ {\varvec{x}}^{p}_0; {\varvec{x}}^{p}_1\varvec{E}; {\varvec{x}}^{p}_2\varvec{E};\dots ; {\varvec{x}}^{p}_N\varvec{E}\right] + \varvec{E}^{pos}, \end{aligned}$$

where $\varvec{E}^{pos}$ is an $(N+1)\times d$ matrix, holding the relative position of each patch in a *d*-dimensional vector.

#### Transformer encoder

A typical transformer model consists of two major parts called encoder and decoder. In this paper, we aim to utilize only the former part. The transformer encoder is where the attention mechanism tries to find the similarities among the $N+1$ patches that arrive at its input. As can be seen in Fig. 4b, there are *L* identical layers of transformer encoder in the $\text {CT-HGR}$ network and each has three separate blocks, named as “Layer Norm”, “Multi-head Self Attention (MSA)” and “MLP”. The $z_0$ sequence of patches that is explained above is first fed to a normalization layer to improve the generalization performance of the model and accelerate the training process^47^. The “Layer Norm block” is then followed by the MSA module, which incorporates *h* parallel blocks (heads) of the scaled dot-product attention (also known as self attention). In the context of self attention, three different vectors *Keys*(*K*), *Queries*(*Q*) and *Values*(*V*) of dimension *d* are employed for each input patch. For computing the self attention metric, the dot product of *Queries* and all the *Keys* are calculated and scaled by 1/$\sqrt{d}$ in order to prevent the dot products from generating very large numbers. This matrix is then, converted into a probability matrix through a *softmax* function and is multiplied to the *Values* to produce the attention metric as follows

3 $$\begin{aligned} Attention = softmax\left( \frac{QK^T}{\sqrt{d}}\right) V. \end{aligned}$$

In the MSA block (Fig. 4c), instead of dealing with *d*-dimensional *Queries*, *Keys* and *Values*, we split them into *h* parallel heads and measure the self attention (Fig. 4d) metric on these heads independently. Finally, after finding the corresponding results for each head, we concatenate them to obtain the *d*-dimensional vectors of patches. As indicated in Fig. 4b, residual paths from the encoder’s input to the output of the MSA block are employed to avoid the gradient vanishing problem. The formulations for the above explanations are as follows

4 $$\begin{aligned} z^{'}_l= & {} MSA(LayerNorm(z_{l-1})) + z_{l-1}, \end{aligned}$$

5 $$\begin{aligned} z_l= & {} MLP(LayerNorm(z^{'}_{l})) + z^{'}_{l}, \end{aligned}$$

where $z_l$ is the* l*th transformer layer’s output and $l=1,\dots , L$. The final output of the transformer encoder is given by

6 $$\begin{aligned} z_L= \left[ z^{p}_{L0}; z^{p}_{L1}; \dots ; z^{p}_{LN}\right] , \end{aligned}$$

where $z^{p}_{Li}$ is the final layer’s output corresponding to the $i^{th}$ patch and $i=1,\dots , N$. As mentioned before, among all the above vector of patches, the $z^{p}_{L0}$ vector matching the class token is chosen for gesture classification. Authors in^48^ claim that the learned features in the sequence of patches will eventually be included in the class token, which has a decisive role in predicting the model’s output. Therefore, $z^{p}_{L0}$ is passed to a linear layer which outputs the predicted gesture’s label as

7 $$\begin{aligned} y_{\text {predicted}} = Linear\left( z^{p}_{L0}\right) . \end{aligned}$$

### Power spectral density (PSD) analysis

One of the experiments we did in this paper is comparing performance of our proposed $\text {CT-HGR}$ architecture with that of the conventional ML and a 3D CNN models. For the former, we design two sets of traditional ML algorithms based on SVMs and LDAs, which are commonly^49–53^ used for hand gesture recognition tasks. In the first experiment and following^49–51^, we trained SVM and LDA models based on the following set of classical features: Root Mean Square (RMS), Zero Crossings (ZC), Slope Sign Change (SSC), and Wave-length (WL). To observe effects of recently proposed feature extraction methods, we did a second experiment based on features introduced in Reference^53^. These features are a rough estimate of the Power Spectral Density (PSD) of the signal by finding an approximate relation between the PSD in the frequency domain and the time-domain signal utilizing characteristics of the Fourier transform and the Parseval’s theorem. According to Parseval’s theorem, the sum of squares of a function is equal to the sum of squares of its Fourier transform, i.e.,

1 $$\begin{aligned} \sum _{j=0}^{N-1} {| {x\left[ {j}\right] } |^{2}} =\frac{1}{N}\sum _{k=0}^{N-1} {| {X\left[ {k}\right] X*\left[ {k}\right] } |} =\sum _{k=0}^{N-1} {P\left[ {k}\right] } \end{aligned}$$

where *x* is the original sEMG signal, *X*, its discrete Fourier transform, $X*$, the conjugate of *X*, *P* is the power spectrum, and terms *j*, *k* are the time and frequency indices, respectively. The utilized set of features are $m_{0}$, $m_{1} - m_{0}$, $m_{2}$, $m_{3} - m_{2}$, and $m_{4}-m_{3}$, which are defined as follows

$$\begin{aligned} m_{0}=\frac{A^{\lambda }}{\lambda },\quad m_{1} =\frac{B^{\lambda }}{\lambda }, \quad \text{ and }\quad m_{2} =\frac{C^{\lambda }}{\lambda } \quad m_{3}=\frac{D^{\lambda }}{\lambda } \quad \text{ and } \quad m_{4} =\frac{E^{\lambda }}{\lambda }, \end{aligned}$$

where

$$\begin{aligned} A= & {} \sqrt{\frac{\sum _{j=0}^{N-1} {| {x\left[ {j}\right] ^{2}} |}}{N}}, \quad B=\sqrt{\frac{\sum _{j=0}^{N-1} {| {\Delta \bullet x\left[ {j}\right] } |^{2}}}{N}}, \quad D = \sqrt{\frac{\sum _{j=0}^{N-1} {| {\Delta ^{2}\bullet x\left[ {j}\right] |^{2}}}}{N}}, \quad C=\sqrt{\frac{\sum _{j=0}^{N-1} {\Delta d_{1}^{2}}}{N}},\\{} & {} \quad \text {and} \quad E=\sqrt{\frac{\sum _{j=0}^{N-1} {\Delta d_{2}^{2}}}{N}}, \end{aligned}$$

where $\Delta \bullet , \Delta ^{2}\bullet$ are the signs for the first and second derivatives and $d_{1}, d_{2}$ are the first and second derivatives of the original sEMG signal.

In the next section, the results corresponding to running conventional ML models using the above-mentioned sets of features are shown. Moreover, we will describe all other various experiments performed in this study and present the obtained results and their explanations in detail.

## Results

We perform several experiments to evaluate performance of the proposed framework under different configurations. In the following, each of the conducted experiments and their corresponding results are presented separately. The implemented models are evaluated on all the 66 gestures of the HD-sEMG dataset performed by 19 healthy subjects. The implementations were developed in the PyTorch framework and the models are trained using an NVIDIA GeForce GTX 1080 Ti GPU.

### Overall performance evaluation under different configurations

**Table 2:** Comparison of classification accuracy and STD for each fold and their average for different window sizes and number of channels ($\text {CT-HGR}$-V1). The accuracy and STD for each fold is averaged over 19 subjects

# Channels	Window size (samples)	Fold1 (%)	Fold2 (%)	Fold3 (%)	Fold4 (%)	Fold5 (%)	Average (%)
32	64	76.85 (±3.83)	89.30 (±2.61)	89.91 (±2.54)	89.62 (±2.67)	85.49 (±3.07)	86.23 (±2.94)
	128	77.21 (±3.56)	89.48 (±2.60)	90.05 (±2.63)	90.00 (±2.61)	85.83 (±2.96)	86.51 (±2.87)
	256	77.63 (±3.50)	90.51 (±2.52)	90.79 (±2.45)	90.99 (±2.42)	86.66 (±2.97)	87.32 (±2.77)
64	64	79.64 (±3.38)	91.92 (±2.41)	92.55 (±2.18)	92.37 (±2.32)	88.16 (±2.77)	88.93 (±2.61)
	128	80.26 (±3.44)	92.32 (±2.27)	92.94 (±2.20)	92.48 (±2.22)	88.46 (±2.77)	89.29 (±2.58)
	256	81.43 (±3.31)	92.89 (±2.15)	93.42 (±2.13)	93.05 (±2.18)	89.29 (±2.69)	90.02 (±2.49)
128	64	82.14 (±3.26)	93.30 (±2.14)	93.75 (±2.08)	93.39 (±2.11)	90.07 (±2.55)	90.53 (±2.43)
	128	82.80 (±3.22)	93.47 (±2.13)	93.98 (±2.03)	93.82 (±2.10)	90.30 (±2.48)	90.87 (±2.39)
	256	83.20 (±3.21)	94.19 (±2.00)	94.25 (±1.97)	94.42 (±1.91)	90.70 (±2.46)	91.35 (±2.31)
	512	83.87 (±3.21)	94.62 (±1.88)	95.26 (±1.80)	94.89 (±1.85)	91.26 (±2.37)	91.98 (±2.22)

**Table 3:** Comparison of classification accuracy and STD for each fold and their average for different window sizes and 128 electrode channels ($\text {CT-HGR}$-V2). The accuracy and STD for each fold is averaged over 19 subjects

# Channels	Window size (samples)	Fold1 (%)	Fold2 (%)	Fold3 (%)	Fold4 (%)	Fold5 (%)	Average (%)
128	64	83.82 (±3.22)	94.03 (±2.02)	94.58 (±1.9)	94.29 (±2.05)	90.84 (±2.58)	91.51 (±2.35)
	128	83.98 (±3.17)	94.09 (±2.00)	94.82 (±1.86)	94.65 (±1.94)	90.89 (±2.45)	91.69 (±2.28)
	256	84.74 (±3.13)	94.60 (±1.92)	95.19 (±1.80)	95.06 (±1.86)	91.59 (±2.44)	92.24 (±2.23)
	512	85.27 (±3.12)	95.55 (±1.70)	95.81 (±1.65)	95.60 (±1.73)	92.16 (±2.32)	92.88 (±2.10)

**Table 4:** The number of learnable parameters for different number of electrodes and window sizes

# Channels	Window size (samples)	# Parameters ($\text {CT-HGR}$-V1)	# Parameters ($\text {CT-HGR}$-V2)	# Parameters (3D CNN)
32	64	46,530	–	–
	128	47,042	–	–
	256	48,066	–	–
64	64	62,914	–	294,914
	128	63,426	–	311,298
	256	64,450	–	319,490
128	64	95,682	273,346	–
	128	96,194	274,370	–
	256	97,218	276,418	–
	512	99,266	280,514	–

In this experiment, we employ 4 different window sizes together with 3 different combination of electrodes of the HD-sEMG dataset and report the achieved accuracy for each of the 5 test folds and the overall averaged accuracy. In the first model, referred to as the $\text {CT-HGR}$-V1, the simplest and smallest $\text {CT-HGR}$ model that gives acceptable results is chosen. The length of windowed signals, in this model, is set to 64, 128, 256 and 512 (31.25, 62.5, 125, 250 ms respectively) with skip step of 32 except for the window size of 512 for which the skip step is set to 64. To measure effects of increasing the number of channels on the performance of the proposed architecture, we consider three different settings using all, half, and 1/4 of the 128 electrodes. In the half mode, electrodes of multiple of 2 and in the 1/4 mode, electrodes of multiple of 4 were chosen. In this regard, we chose one electrode out of four adjacent electrodes to make sure that the utilized electrodes still cover the whole recorded area and the only thing that changes is the distance among the chosen electrodes. In such a scenario (which intuitively speaking can be interpreted as an unbiased way of choosing the electrodes), we make sure that we do not miss much of the information that high density grids usually provide and the model do not lose its generalizability when being fed with the data from fewer number of electrode channels. As stated previously, the number of horizontal electrode channels in the $\text {CT-HGR}$’s input is 4, 8, and 16 while the number of vertical channels is 8. Regarding the hyperparameters of the model, the model’s (embedding) dimension is 64, and the patch size is set to (8, 4), (8, 8), and (8, 16) for 32, 64, and 128 number of channels, respectively. The $\text {CT-HGR}$-V1 model contains only 1 transformer layer and 8 heads. The MLP block’s hidden size is set to 64, the same as its input size. The $\text {CT-HGR}$-V1 model is trained with 20 epochs and batch size of 128 for each subject independently. The optimization method used is Adam with $\beta 1 = 0.9$ and $\beta 2 = 0.999$ parameters, learning rate of 0.0001 and weight decay of 0.001. Learning rate annealing is deployed after the first 10 epochs for faster convergence. The cross-entropy loss function is considered as the objective function. Table 2 represents the acquired accuracy and standard deviation (STD) for each individual window size and number of channels. It is worth noting that the 512 window size is only tested with the whole electrode channels of the dataset to indicate the potential best performance of the network.

**Figure 5:**
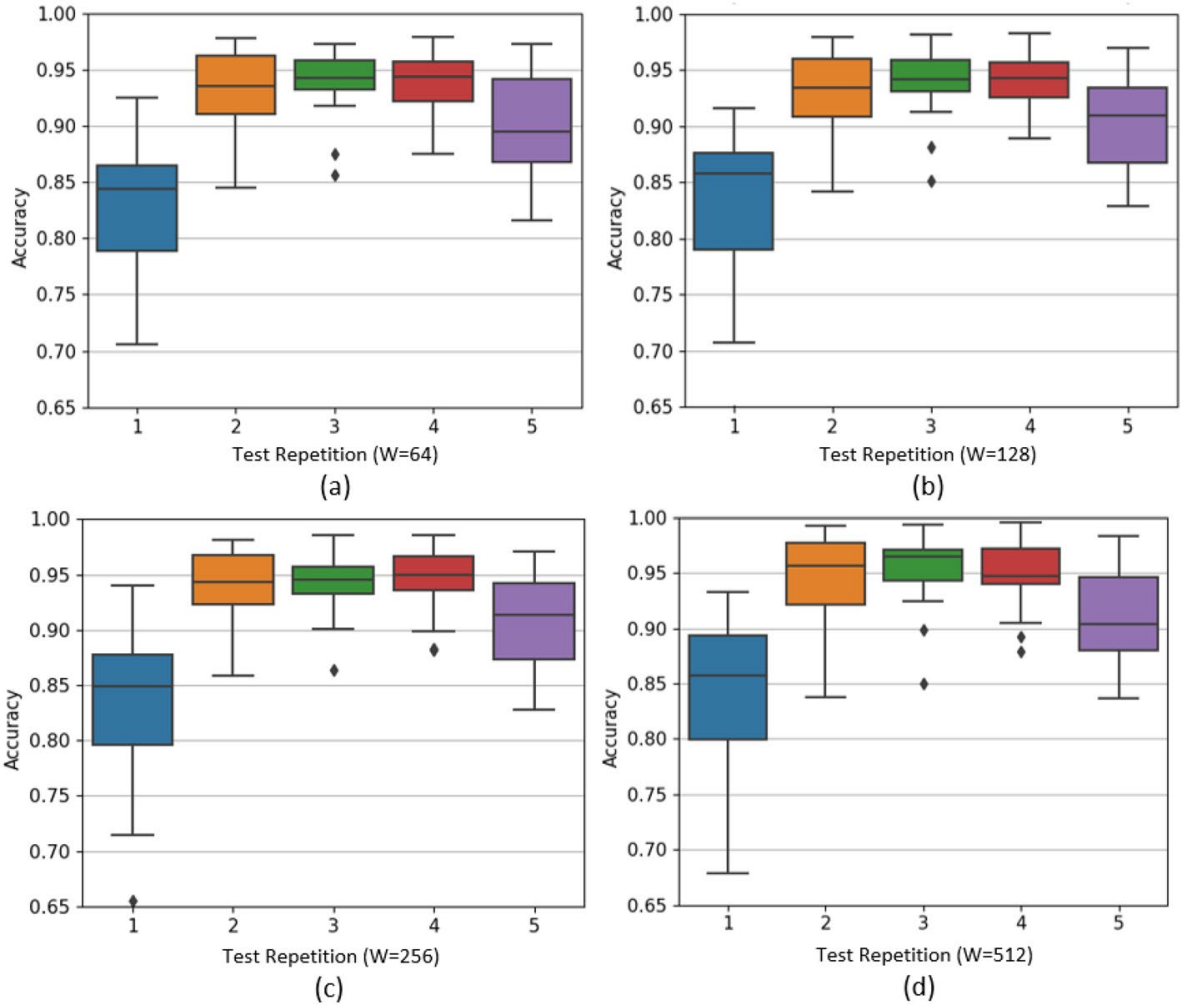
Comparison of the accuracy $\text {CT-HGR}$-V1 obtains for each fold and window sizes of (**a**) $W=64$ (**b**) $W=128$ (**c**) $W=256$ and (**d**) $W=512$. The number of utilized electrode channels in these plots is 128.

**Figure 6:**
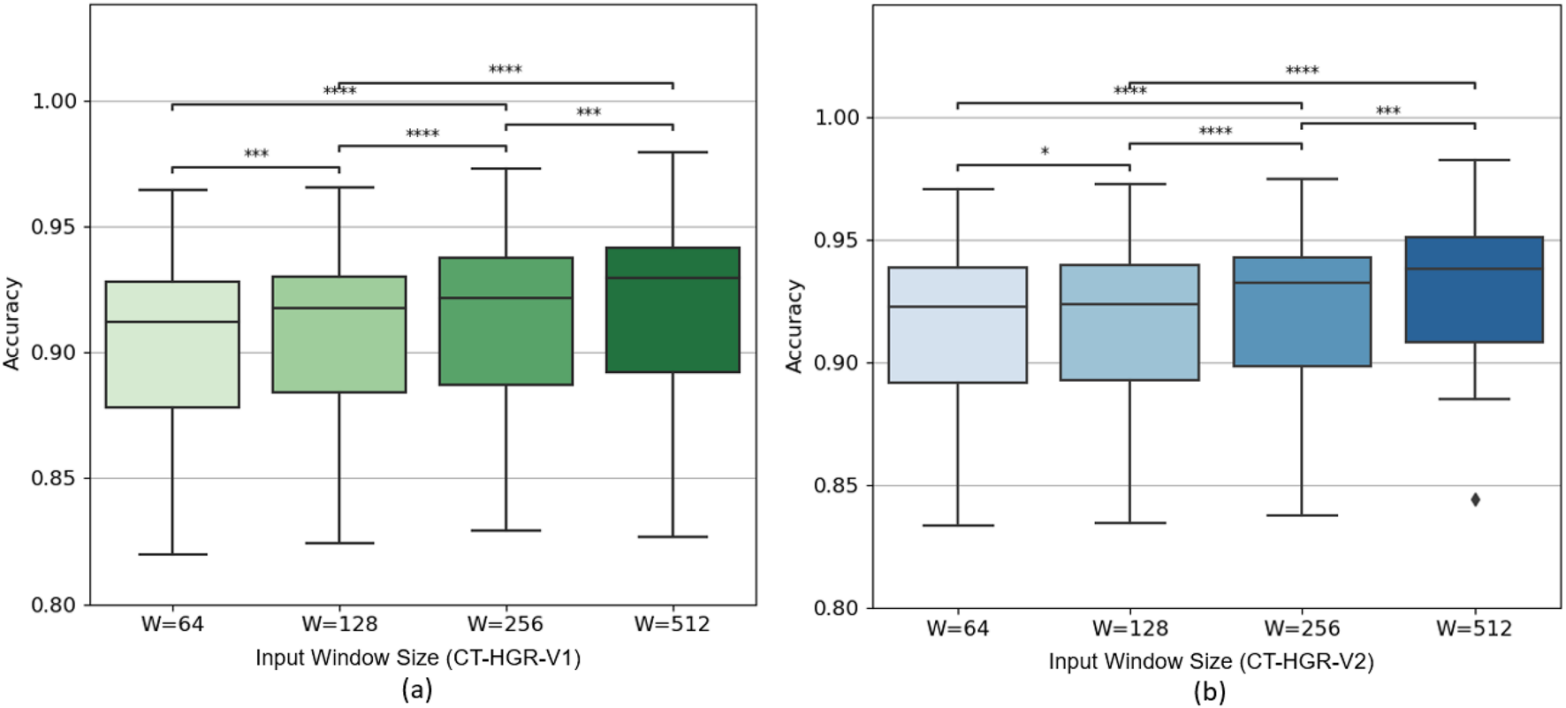
Statistical analysis of training over different window sizes, i.e., $W=64$, $W=128$, $W=256$, and $W=512$ for (**a**) $\text {CT-HGR}$-V1, and (**b**) $\text {CT-HGR}$-V2. The box plots are drawn based on the InterQuartile Range (IQR) of the accuracy for all the subjects and all the electrodes.

A second variant of the $\text {CT-HGR}$ model, referred to as $\text {CT-HGR}$-V2, is also tested where the model’s dimension and the number of hidden layers in the MLP layer are twice those of $\text {CT-HGR}$-V1. We apply the $\text {CT-HGR}$-V2 model on the data samples derived from the whole 128 electrodes to compare it with the last 4 rows of Table 2. The results are shown in Table 3. Table 4 illustrates the number of learnable parameters for each window size and number of channels in both models. Fig. 5 demonstrates the box plots for the accuracy of $\text {CT-HGR}$-V1 obtained for each individual fold and different window sizes from $W=64$ to $W=512$ (Fig. 5a–d. The box plots are drawn based on the InterQuartile Range (IQR) of accuracy for 19 subjects when all the 128 electrodes are included in the experiment. The black horizontal line represents the median accuracy for each fold. In Fig. 6, the Wilcoxon signed rank test is applied for $\text {CT-HGR}$-V1 and $\text {CT-HGR}$-V2 separately when the number of channels is fixed at 128. The box plots show the IQR for each window size that decreases minimally from $\text {CT-HGR}$-V1 to $\text {CT-HGR}$-V2. The Wilcoxon test’s *p*-value annotations in Fig. 6 are as follows:

- ns: $$5.00e-02< p <= 1.00e+00$$
*: $$1.00e-02< p <= 5.00e-02$$
**: $$1.00e-03< p <= 1.00e-02$$
***: $$1.00e-04< p <= 1.00e-03$$
****: $$p <= 1.00e-04$$

Although the average accuracy does not change significantly, the STD in $\text {CT-HGR}$-V2 with $W=512$ declines significantly compared to $\text {CT-HGR}$-V1.

**Figure 7:**
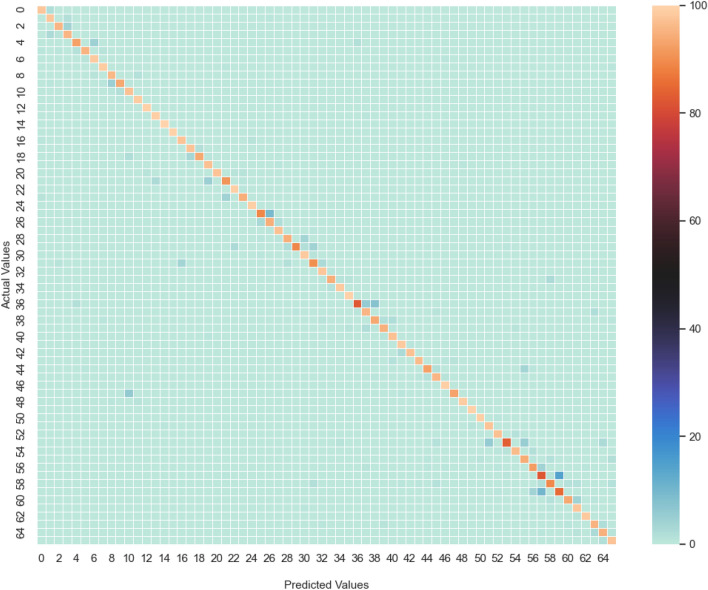
Average confusion matrix of Model $\text {CT-HGR}$-V1 with $W=512$ and 128 number of electrodes over repetition 3 of 19 subjects.

**Figure 8:**
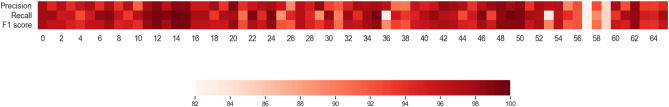
Representation of Precision, Recall and F1 Score of Model $\text {CT-HGR}$-V1 with $W=512$ and 128 number of electrodes over repetition 3 of 19 subjects. These measures are obtained from the confusion matrix of Fig. 7 and shown for each class separately.

**Figure 9:**
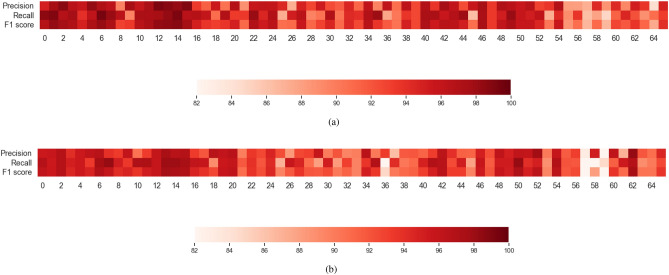
Representation of Precision, Recall and F1 Score with $W=256$ and 64 number of electrodes over repetition 3 of all 19 subjects: (**a**) Model SVM-V1. (**b**) $\text {CT-HGR}$-V1.

**Table 5:** Average Precision, Recall and F1 Score of Model $\text {CT-HGR}$-V1 with $W = 512$ and 128 number of electrodes over repetition 3 of all 19 subjects

Class #	Precision(%)	Recall(%)	F1 Score(%)	Class #	Precision(%)	Recall(%)	F1 Score(%)
1	97.6 (±3.8)	97.8 (±5.3)	97.7 (±3.7)	34	97.5 (±4.8)	94.2 (±12.3)	95.8 (±8.3)
2	94.5 (±7.2)	97.9 (±9.7)	96.1 (±7.0)	35	97.2 (±4.4)	98.1 (±5.4)	97.7 (±3.9)
3	96.8 (±9.2)	95.4 (±15.3)	96.1 (±13.0)	36	97.5 (±4.7)	99.4 (±1.4)	98.4 (±2.5)
4	94.1 (±12.0)	95.6 (±9.4)	94.8 (±9.6)	37	96.7 (±8.7)	82.6 (±27.8)	89.1 (±23.2)
5	95.9 (±23.6)	92.5 (±23.7)	94.2 (±23.5)	38	90.6 (±12.7)	95.4 (±10.0)	92.9 (±9.9)
6	97.4 (±3.5)	95.2 (±11.2)	96.3 (±6.9)	39	90.4 (±13.7)	93.5 (±13.1)	92.0 (±12.7)
7	94.1 (±12.1)	98.4 (±3.5)	96.2 (±8.3)	40	96.0 (±7.3)	94.7 (±12.8)	95.4 (±10.4)
8	97.2 (±6.6)	98.7 (±2.1)	97.9 (±4.3)	41	94.8 (±6.7)	97.2 (±5.9)	96.0 (±5.3)
9	93.6 (±8.7)	95.9 (±9.1)	94.8 (±8.3)	42	95.6 (±7.8)	98.3 (±2.7)	96.9 (±4.8)
10	96.9 (±7.5)	93.4 (±11.4)	95.1 (±8.3)	43	98.3 (±2.6)	96.9 (±10.3)	97.6 (±6.9)
11	91.4 (±12.0)	96.9 (±12.7)	94.1 (±11.1)	44	98.0 (±3.3)	96.1 (±12.6)	97.0 (±8.8)
12	97.6 (±5.7)	98.7 (±2.9)	98.1 (±3.3)	45	97.4 (±8.3)	92.5 (±16.9)	94.9 (±13.3)
13	98.8 (±2.7)	99.4 (±1.2)	99.1 (±1.4)	46	93.7 (±9.1)	95.4 (±8.7)	94.5 (±8.2)
14	96.6 (±6.5)	99.0 (±2.0)	97.8 (±3.8)	47	96.9 (±4.5)	98.9 (±1.8)	97.9 (±2.6)
15	98.9 (±2.2)	99.7 (±1.0)	99.3 (±1.2)	48	98.0 (±22.4)	92.8 (±22.2)	95.3 (±22.1)
16	98.7 (±2.0)	99.5 (±1.5)	99.1 (±1.2)	49	98.7 (±2.0)	98.5 (±3.7)	98.6 (±2.3)
17	95.5 (±9.0)	97.5 (±4.8)	96.5 (±6.0)	50	98.0 (±3.5)	99.5 (±1.1)	98.7 (±1.9)
18	95.4 (±6.6)	97.4 (±9.5)	96.4 (±7.1)	51	98.6 (±2.8)	99.0 (±1.7)	98.8 (±1.7)
19	97.7 (±6.5)	93.5 (±9.7)	95.5 (±6.9)	52	93.7 (±11.4)	97.5 (±4.6)	95.6 (±8.1)
20	93.8 (±12.5)	97.2 (±6.2)	95.4 (±10.0)	53	97.6 (±3.9)	97.0 (±5.8)	97.3 (±4.4)
21	99.0 (±1.4)	97.5 (±5.0)	98.2 (±3.1)	54	96.6 (±19.1)	83.5 (±27.3)	89.6 (±26.3)
22	93.9 (±22.4)	90.7 (±23.1)	92.3 (±22.2)	55	97.2 (±7.6)	96.2 (±11.0)	96.7 (±9.2)
23	95.5 (±7.6)	99.1 (±3.0)	97.3 (±4.7)	56	89.3 (±15.3)	94.0 (±11.5)	91.6 (±12.7)
24	96.9 (±3.3)	94.2 (±12.4)	95.5 (±8.1)	57	92.3 (±14.2)	91.9 (±11.1)	92.1 (±12.6)
25	97.5 (±4.3)	99.1 (±1.2)	98.3 (±2.3)	58	82.2 (±15.2)	82.4 (±27.9)	82.3 (±25.6)
26	95.5 (±14.0)	88.8 (±25.4)	92.0 (±23.6)	59	92.5 (±11.5)	89.1 (±19.5)	90.7 (±15.7)
27	89.0 (±15.8)	94.5 (±10.0)	91.6 (±12.3)	60	84.6 (±15.3)	84.8 (±24.9)	84.7 (±20.5)
28	96.6 (±5.7)	97.0 (±5.1)	96.8 (±4.4)	61	97.6 (±4.0)	93.7 (±17.1)	95.6 (±13.2)
29	95.1 (±5.6)	94.5 (±14.4)	94.8 (±10.5)	62	92.3 (±11.5)	97.7 (±6.6)	94.9 (±8.1)
30	98.4 (±3.1)	88.8 (±19.6)	93.4 (±15.0)	63	98.5 (±2.7)	98.4 (±4.9)	98.5 (±2.9)
31	93.0 (±9.6)	98.2 (±2.4)	95.5 (±5.6)	64	93.3 (±8.8)	95.2 (±8.4)	94.2 (±7.0)
32	91.8 (±23.4)	89.9 (±25.4)	90.8 (±23.9)	65	93.2 (±8.2)	94.8 (±8.2)	94.0 (±6.7)
33	94.7 (±10.6)	98.0 (±3.5)	96.3 (±7.2)	66	94.4 (±9.2)	97.5 (±6.7)	96.0 (±6.6)

The gestures in the HD-sEMG dataset are ordered according to their DoF and similarity in performance. The simple 1 DoF gestures are labeled from 1 to 16, 2 DoF gestures are from 17 to 57 and the most complex ones are from 58 to 66. To be more specific, the confusion matrices for Model $\text {CT-HGR}$-V1 with $W=512$ and 128 number of channels are obtained for repetition 3 of all the subjects. The matrices are summed and normalized row-wise. The final confusion matrix is shown in Fig. 7. The diagonal values show the average accuracy acquired for each hand gesture among 19 subjects. The average accuracy for most of the gestures is above 94%. The density of the non-zero elements in Fig. 7 is utmost near the diagonal, which implies that the possibility of the network making mistakes in gesture classification is higher in gestures that have the same DoF and are performed similarly. Fig. 8 represents precision, recall, and F1 score associated with Model $\text {CT-HGR}$-V1 for each gesture based on the confusion matrix shown in Fig. 7. This figure is included to provide the readers with a better sense of the gestures for which the above metrics were significantly high or low. Corresponding results for each gesture are illustrated in Table 5 and the average Matthews Correlation Coefficient (MCC) measure among all the subjects is calculated as 95.2%.

### Comparisons with a conventional ML and a 3D convolutional model

In the first part of this sub-section, we design two sets of traditional ML algorithms based on SVMs and LDAs, which are commonly^49–53^ used for hand gesture recognition tasks. In the first experiment and following^49–51^, we trained SVM and LDA models based on the following set of classical features: Root Mean Square (RMS), Zero Crossings (ZC), Slope Sign Change (SSC), and Wave-length (WL). This experiment resulted in two models called SVM-V2 and LDA-V2. There are, however, some promising new feature extraction methods proposed in the recent literature^52–56^. To observe effects of recently proposed feature extraction methods, we did a second experiment based on features introduced in Reference^53^. These features are a rough estimate of the Power Spectral Density (PSD) of the signal by finding an approximate relation between the PSD in the frequency domain and the time-domain signal utilizing characteristics of the Fourier transform and the Parseval’s theorem. The procedures on how to extract these features from raw HD-sEMG data is explained in Sect. "Power spectral density (PSD) analysis".

**Table 6:** Comparison of classification accuracy and STD for different window sizes and 64 electrode channels using $\text {CT-HGR}$-V1, 3D CNN, SVM-V1, SVM-V2, LDA-V1, and LDA-V2 models. The accuracy and STD are averaged over all the 5 folds and 19 subjects

# Channels	Window size (samples)	$\text {CT-HGR}$-V1 (%)	3D CNN (%)	SVM-V1 (%)	SVM-V2 (%)	LDA-V1 (%)	LDA-V2 (%)
64	64	88.93 (±2.61)	86.15 (±2.95)	86.01 (±7.05)	74.49 (±11.56)	83.05 (±7.35)	71.40 (±12.45)
	128	89.29 (±2.58)	86.68 (±2.85)	89.95 (±5.19)	83.4 (±8.66)	87.97 (±5.38)	81.10 (±9.59)
	256	90.02 (±2.49)	87.45 (±2.77)	90.71 (±4.88)	87.77 (±5.84)	90.85 (±4.46)	86.72 (±7.37)

**Figure 10:**
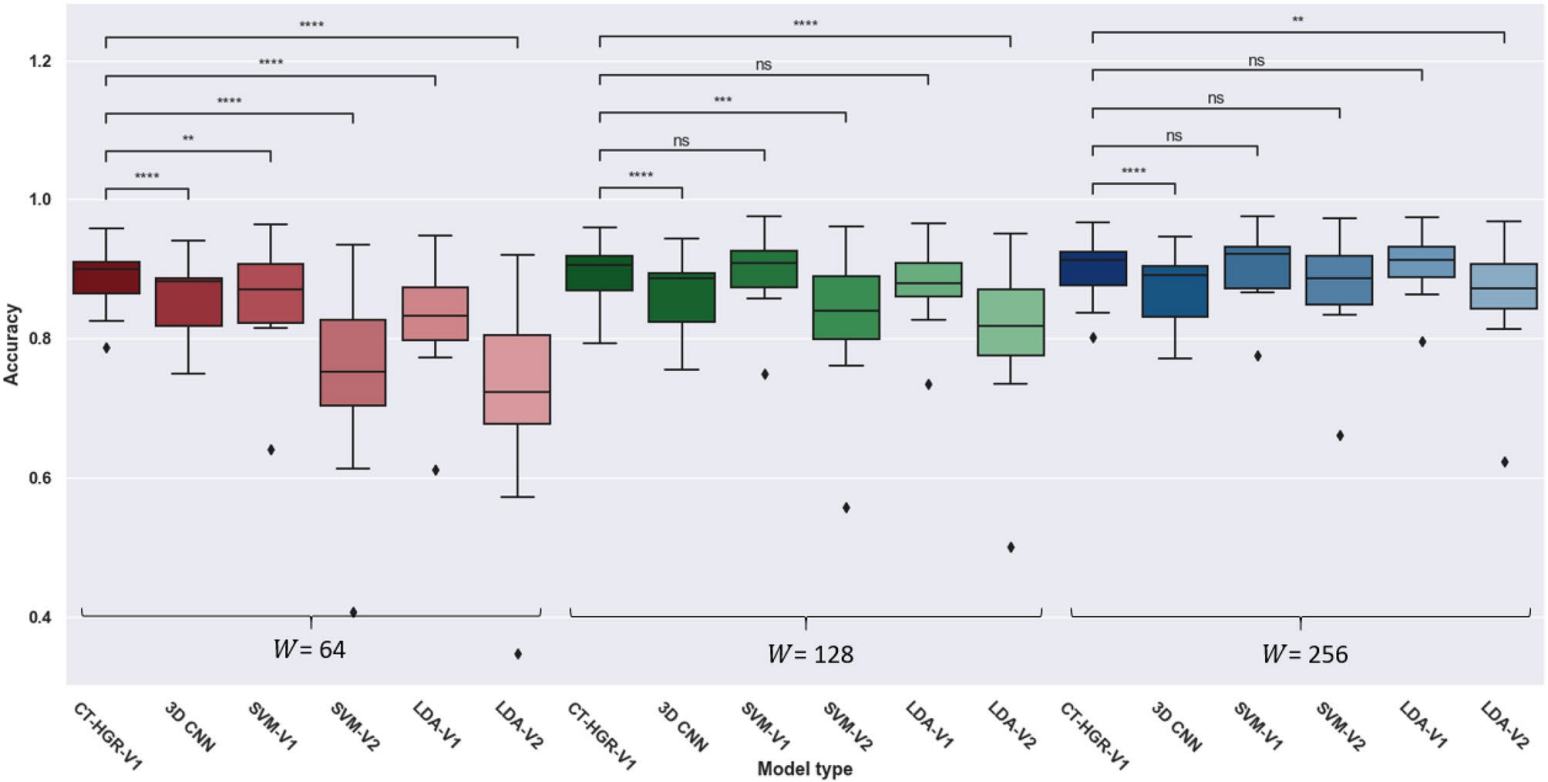
Box plots and IQR of $\text {CT-HGR}$-V1, 3D CNN, SVM-V1, SVM-V2, LDA-V1, and LDA-V2 for different window sizes ($W=64$ $W=128$ and $W=256$) and 64 number of channels.

In the second part, we implement a 3D CNN model that is originally utilized for video-based hand gesture recognition tasks^57^ and is found effective by authors in^58^ to be applied on HD-sEMG datasets as they resemble video data in having one dimension in time and two dimensions in space. Therefore, in spite of a typical 2D CNN model, a 3D CNN architecture is able to extract both the temporal and spatial features in HD-sEMG datasets. The 3D signals of shape $W\times N_{ch}\times N_{cv}$ go through the 3D CNN architecture that has two consecutive 3D CNN layers with 16 and 32 respective filters of size (5, 3, 3), each followed by a GELU activation function, a dropout and a max pooling layer. Then, two fully connected (FC) layers of size 256 and 128 are deployed before the output layers which consists of an MLP head similar to the one used in our $\text {CT-HGR}$ models followed by a *softmax* function for classification. The other hyperparameters of the network are set similar to those of the $\text {CT-HGR}$ model. The stride values in both 3D CNN layers are 1. Table 6 shows the acquired results for the ML and 3D CNN models in which the number of channels in the dataset is set to 64. For the case of ML models, Fig. 9 compares precision, recall, and F1 score metrics obtained from the best performing ML model (SVM-V1) with that of our proposed $\text {CT-HGR}$-V1 with the same settings ($W=256$ and 64 number of electrode channels). The average MCC measure for SVM-V1 is calculated as 94.2% and for $\text {CT-HGR}$-V1 as 93.1%. Fig. 10 shows the box plots and the results of Wilcoxon signed rank statistical test that is conducted for comparing $\text {CT-HGR}$-V1, 3D CNN, SVM-V1, SVM-V2, LDA-V1, LDA-V2 model’s performance accuracy on 19 subjects. In this experiment, the window sizes for all the models are changed ($W=64$, $W=128$ and $W=256$), but the number of channels is fixed at 64. Therefore, only the models accepting the same window size as the input are compared to assess the discrepancy between two different models with the same input data.

**Table 7:** Comparison of train time, test time, and the maximum allocated memory for $W=256$ and 64 electrode channels using $\text {CT-HGR}$-V1, 3D CNN, SVM-V1, SVM-V2, LDA-V1, and LDA-V2 models

# Channels	Window size (samples)	Parameter	$\text {CT-HGR}$-V1	3D CNN	SVM-V1	SVM-V2	LDA-V1	LDA-V2
64	256	Train time (s)	382.9	1228.9	203.2	187.4	149.3	160
		Test time (s)	69	8	237.3	374.7	31.6	36.2
		Memory (GB)	14.80	14.81	40.60	21.47	40.60	21.47

When it comes to evaluation of the computational cost for DL models, the ultimate objective is to measure the needed amount of resources in training and inference. Computational cost can be measured in a variety of ways, among which time, memory and number of Floating Point Operations (FLOPs) are the common metrics. To evaluate computational cost of the proposed framework, in addition to the number of trainable parameters shown in Fig. 4, we have calculated the train time, test time and maximum allocated memory for each of the $\text {CT-HGR}$-V1, 3D CNN, SVM-V1, SVM-V2, LDA-V1, and LDA-V2 models, which are shown in Table 7. Please note that the train/test times reported in Table 7 correspond to the whole train/test data containing all segments of 256-sample windows. Considering 4 repetitions in the train set and 1 repetition in the test set for each subject, we have approximately 73, 000 and 18, 000 samples in the train and test set, respectively. This means that, $\text {CT-HGR}$-V1 for which the test time is reported as 69 seconds, needs 3.8 ms to predict each 256-sample window’s corresponding gesture. We should point out that different factors, such as the GPU memory, how the code is organized, and the utilized batch size, can affect test time specifically in the small scale of each window size. It is also worth noting that memory bandwidth is considered instead of FLOPs because on existing hardware architectures, a single memory access is much slower than a single computation.

### Performance evaluation based on shuffled data

**Table 8:** Accuracy and STD for the shuffled dataset of all the 5 repetitions and different window sizes ($\text {CT-HGR}$-V1).

# Channels	Window size (samples)	# Avg accuracy (%)
64	64	98.05 (±1.19)
	128	98.43 (±1.05)
	256	98.79 (±0.96)

In the previous sub-sections, a 5-fold cross-validation technique was applied on the HD-sEMG dataset in which the test set (repetition) is entirely unseen and is not included in the train set (repetitions). However, another approach followed in the literature^9,59^ to split the train/test sets is to shuffle the whole dataset with *n* repetitions and assign an arbitrary portion to the train set and the remaining to the test set. Along the same line, in some of the previous works^41,42,44^ either the train/test splits were not specified or it was mentioned that data for each subject was shuffled and then randomly divided into train/test sets. Intuitively speaking, by shuffling the dataset across different repetitions, the model can better catch variations of the underlying signals and provide improved performance. In practice, the overall objective would be to have a generalizable model that works under different conditions as such one can acquire different repetitions and train the model over all to boost the performance. To observe effects of such a training approach on the overall achievable accuracy, we have decided to include such an experiment by shuffling the dataset. The results and observations are on a par with those reported in the aforementioned reference^41,42,44^.The obtained average accuracy over 19 participants using 64, 128, 256 window sizes using the hyperparameters of $\text {CT-HGR}$-V1 are summarized in Table 8.

### Instantaneous performance evaluation

**Table 9:** Accuracy and STD of each fold and their average for instantaneous training.

# Channels	Window size (samples)	Fold1 (%)	Fold2 (%)	Fold3 (%)	Fold4 (%)	Fold5 (%)	Average (%)
64	1	80.02 (±3.45)	92.33 (±2.27)	92.47 (±2.26)	92.16 (±2.31)	88.69 (±2.74)	89.13 (±2.61)

In this sub-section, our objective is to assess the functionality of the proposed framework on instant HD-sEMG data points. In other words, we consider window size of only 1 sample as the input to our model, which requires no patching. We set the number of electrodes to 64. The hyperparameters used in this experiment are the same as those used for $\text {CT-HGR}$-V1. The accuracy results are presented in Table 9.

### Evaluation of a hybrid model based on raw HD-sEMG and extracted MUAPs

**Figure 11:**
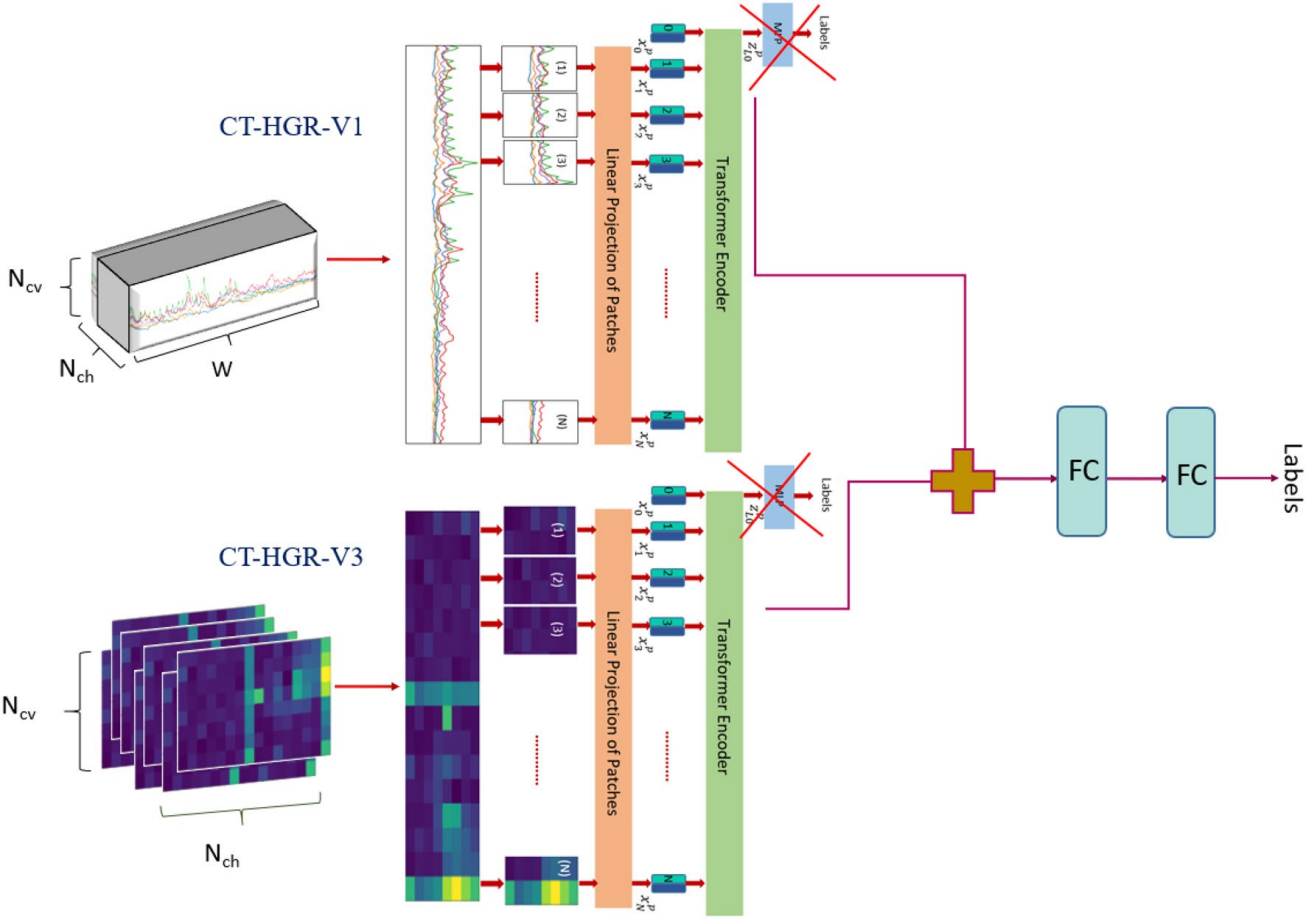
The fused $\text {CT-HGR}$ framework. In the first stage, the ViT-based models in the Macro and Micro paths are trained based on 3D, HD-sEMG and 2D, peak-to-peak MUAP images, respectively. In the second stage, the Micro and Micro weights are frozen (not being updated with gradient descent during training). The final Micro and Macro class tokens are concatenated and converted to a 1, 024-dimensional feature vector, which is fed to a series of FC layers for gesture classification.

In this sub-section, we present the results of fusing $\text {CT-HGR}$-V1 with a third variant of the $\text {CT-HGR}$ called $\text {CT-HGR}$-V3 that works based on the extracted MUAPs from raw HD-sEMG signals. More specifically, $\text {CT-HGR}$-V3 uses HD-sEMG decomposition to extract microscopic neural drive information from HD-sEMG signals for hand gesture recognition. Considering multi-channel sEMG signals as a convolutive mixture of a set of impulse functions known as the Motor Unit Spike Trains (MUSTs) of each MU^60^, sEMG decomposition refers to a set of Blind Source Separation (BSS)^61^ methods that extract discharge timings of motor neuron action potentials from raw HD-sEMG data. Single motor neuron action potentials are summed to form MUAPs that are in charge of converting neural drive information to hand movements^62^. Motor unit discharge timings, also known as MUSTs, represent sparse estimations of the MU activation times with the same sampling frequency and time interval as the raw HD-sEMG signals^63^. HD-sEMG signals can be modelled as a spatio-temporal convolution of MUSTs, which provide an exact physiological description of how each hand movement is encoded at neurospinal level^64^. Thus, MUSTs are of trustworthy and discernible information on the generation details of different hand gestures, as such they are adopted in $\text {CT-HGR}$-V3 for hand gesture recognition.

Generally speaking, for extracting MUSTs, among the existing BSS approaches^60^ suggested for HD-sEMG decomposition, gradient Convolution Kernel Compensation (gCKC)^65,66^ and fast Independent Component Analysis (fastICA)^67^ are of great prominence and are frequently used in the literature. To achieve better accuracy, the utilized BSS algorithm^60^ is a combination of gCKC^65,66^ and fastICA^67^. Detailed explanation of such an integrated BSS algorithm can be found in^60^. In this method, the number of extracted sources is dependent on the following two different parameters that are determined before initiating the algorithm: (i) The number of iterations of gCKC and fastICA algorithms in which a new MU is found, and; (ii) The silhouette threshold, which determines whether the extracted MU is of high quality to be accepted or ignored. As stated in^68,69^, the activation level/area of MUs in limb muscles is highly variable across different hand gestures. Accordingly, if the peak-to-peak values of MUAPs for each MU and all the channels are calculated, a set of 2D images can be acquired, which have a predictable pattern among different hand gestures. Therefore, after extracting the MUSTs of HD-sEMG signals, the corresponding MUAPs are found using Spike-Triggered Averaging (STA) method^69^ with an averaging window of 20 samples. In this approach, for each MUST (each extracted MU), $N_{ch}\times N_{cv}$ MUAPs of length 20 are found. Then, the peak-to-peak values of the MUAPs are calculated and a 2D image of shape $N_{ch}\times N_{cv}$ is constructed for each MU. We should point out that the temporal profile of MUAPs obtained from MUSTs encode information about MU recruitments and the temporal profile of the EMG recordings. Therefore, using sliding windows for extraction of MUSTs informs us about the most current profile of the active MUs, their recruitments, and how much they are involved in each stage of performing the hand gestures.

The fused variant of the $\text {CT-HGR}$ is designed to simultaneously extract a set of temporal and spatial features from HD-sEMG signals through its two independent ViT-based parallel paths, i.e., the *Macro Path* and the *Micro Path*. The former is the $\text {CT-HGR}$-V1 that accepts raw HD-sEMG signals as input, while the latter is the $\text {CT-HGR}$-V3 fed with the peak-to-peak values of the extracted MUAPs of each source. A fusion path, structured in series to the parallel ones and consisting of FC layers, then combines extracted temporal and spatial features for final classification. Fig. 11 illustrates the overall hybrid architecture of the fused model. In particular, the Macro Path extracts both temporal and spatial features of HD-sEMG signals as it is fed with time-series raw HD-sEMG signal that are variable both in terms of time and space. However, the Micro Path can extract another set of spatial features from peak-to-peak values of MUAPs that are variable in space.

In our experiments, the number of iterations (Item (i)) is set to 7 and the silhouette measure (Item (ii)) is set to 0.92, therefore, depending on the quality of the extracted MUSTs, a maximum of 7 sources are estimated for each windowed signal. Therefore, each windowed signal of shape $W\times N_{ch}\times N_{cv}$ is of maximum 7 MUs that retain various activation levels for each electrode channel. These 2D images are considered as new input data to the $\text {CT-HGR}$-V3. Thus, according to Fig. 11, for each windowed signal that is fed to $\text {CT-HGR}$-V1, a maximum of 7 peak-to-peak MUAPs are created and fed to $\text {CT-HGR}$-V3. After training $\text {CT-HGR}$-V1 and V3 independently, the models’ weights are frozen, i.e., are kept constant (not being updated with gradient descent during training) and the final classification linear layer is removed for both models. Then, the final class tokens of $\text {CT-HGR}$-V1 and $\text {CT-HGR}$-V3 are joined together and fed to a FC layer for final classification. In this way, the hybrid model decides based on raw HD-sEMG signals as well as peak-to-peak images of MUAPs obtained for each MU independently. The $\text {CT-HGR}$-V3’s hyperparameters are set as follows: For both $\text {CT-HGR}$-V1 and V3, HD-sEMG data is divided into windows of shape (512,8,16) with skip step of 256. Therefore, the image size and the number of input channels for 2D images are set to ($8\times 16$), and 1, respectively. For each peak-to-peak image, we considered 2 patches by setting patch size to ($8\times 8$). The model’s embedding dimension (*d*) and number of heads is the same as the two previous models. The optimization algorithm is Adam with learning rate of 0.0003 and weight decay of 0.001. Each batch has 64 data samples and the model is trained through 50 epochs. Table 10 compares accuracy and STD for $\text {CT-HGR}$-V1, $\text {CT-HGR}$-V3 and their fused model for each fold. It is worth mentioning that authors in^70^ have adopted a quite similar approach to ours by combining activations of individual DoFs (obtained from decomposed MUSTs) with residual HD-sEMG signals for predicting wrist DoF angles using a linear regression method. The main distinctions between the two methods are as follows: (i) The method of^70^ focuses on predicting DoF angles in wrist kinematics and not gesture recognition, and (ii) Considered combining residual HD-sEMG signals with DoF activations, which is a different concept from combining peak-to-peak MUAPs with original HD-sEMG signals.

**Table 10:** Comparison of classification accuracy and STD for each fold and their average for each of the 3 models. The accuracy and STD for each fold is averaged over 19 participants

Model Name	Fold1 (%)	Fold2 (%)	Fold3 (%)	Fold4 (%)	Fold5 (%)	Average (%)
$\text {CT-HGR}$-V1	79.92 (±3.39)	91.43 (±2.48)	93.84 (±2.05)	92.57 (±2.28)	88.96 (±2.83)	89.34 (±2.61)
$\text {CT-HGR}$-V3	81.53 (±3.45)	88.03 (±2.66)	89.63 (±2.39)	89.11 (±4.02)	84.92 (±2.97)	86.64 (±3.10)
Fused	89.38 (±2.88)	96.86 (±1.82)	96.82 (±1.75)	96.65 (±2.75)	94.61 (±1.90)	94.86 (±2.22)

### Comparison with other works on the utilized dataset

In this section, we compare our proposed $\text {CT-HGR}$ model with 4 other works^41–44^ that proposed ML/DL methods for hand gesture recognition based on the same dataset utilized in this study.

Sun, *et al.*^43^ proposed three different CNN-based models for hand gesture recognition with 1D, 2D and 3D convolutional layers that are applied on both transient and steady phases of HD-sEMG data. In our study and differently from^43^, we jointly considered the transient and steady phases of the sEMG signals when providing the input to the model, therefore, data distribution should be different. We, however, compared our results with their steady phase as there is more similarity between these two types in comparison to the transient phase. Using a window size of 200 ms, all the 128 electrode channels, and the same 5-fold cross validation technique as we implemented, the maximum median accuracy obtained by the model of^43^ is 84.6% whereas the proposed framework obtained 91.98% accuracy for 250 ms window and 128 electrode channels. In^41^, a similar study to ours is conducted by changing the window size and the number of channels to evaluate their effect on the performance of the model. In this paper, 5 time-domain features of the signal along with sixth-order autoregressive coefficients are extracted and given to an LDA model. Average accuracy of 81.39% is obtained for the window size of 32 ms when 32 channels were used. The accuracy increases to 91.5% for the same window size with 128 channels. It finally reaches 96.14% for the 256 ms window and 128 channels with minimum STD of 3.82%. We should note that autoregressive coefficient extraction could be a time-consuming process for HD-sEMG data potentially slowing the learning process. Along a similar path, Reference^44^ introduced a new feature extraction approach using Wavelet Scattering Transform, applied an SVM model on the extracted features and compared their results with that of^41^. The results show an increase in the accuracy for different window sizes and 128 electrode channels which is $\approx$ 94% and 97.2% for 32 ms and 256 ms window sizes, respectively. We should note that in these works, the utilized method for splitting the train/test data is not explicitly specified. A Graph Neural Network approach is adopted in^42^ with window sizes of 65 samples using 128 channels resulting in the average accuracy of 91.25% with STD of 4.92%. Using the same setting, we acquired accuracy of 90.53% and STD of 2.43% with $\text {CT-HGR}$-V1 and 91.51% and STD of 2.35% with $\text {CT-HGR}$-V2. When it comes to train/test datasets, it is mentioned in^42^ that data for each subject was shuffled and then randomly divided into train/test sets. Table 11 represents the average accuracies obtained by the above-mentioned papers and the settings they utilized to assess their performance. If the STD and train/test split is not mentioned in the paper, “NA” (Not Applicable) is shown.

**Table 11:** Comparison of classification accuracy and STD obtained by the other works on our utilized dataset with $\text {CT-HGR}$-V1 and $\text {CT-HGR}$-V2

Reference	Window size (ms)	# Channels	Accuracy (%)	Train/Test Split
Ref^43^	200	128	84.6 (NA)	5-fold Cross Validation
$\text {CT-HGR}$-V1	250	128	91.98 (±2.22)	5-fold Cross Validation
$\text {CT-HGR}$-V2	250	128	92.88 (±2.10)	5-fold Cross Validation
Ref^41^	32	32	81.39 (±10.77)	NA
$\text {CT-HGR}$-V1	31.25	32	86.23 (±2.94)	5-fold Cross Validation
Ref^41^	256	128	96.14 (±4.67)	NA
$\text {CT-HGR}$-V1	250	128	91.98 (±2.22)	5-fold Cross Validation
$\text {CT-HGR}$-V2	250	128	92.88 (±2.10)	5-fold Cross Validation
Ref^42^	31.7	128	91.25 (±4.92)	NA
$\text {CT-HGR}$-V1	31.25	128	90.53 (±2.43)	5-fold Cross Validation
$\text {CT-HGR}$-V2	31.25	128	91.51 (±2.35)	5-fold Cross Validation
Ref^44^	32	128	94 (NA)	NA
$\text {CT-HGR}$-V1	31.25	128	90.53 (±2.43)	5-fold Cross Validation
$\text {CT-HGR}$-V2	31.25	128	91.51 (±2.35)	5-fold Cross Validation
Ref^44^	256	128	97.2 (NA)	NA
$\text {CT-HGR}$-V1	250	128	91.98 (±2.22)	5-fold Cross Validation
$\text {CT-HGR}$-V2	250	128	92.88 (±2.10)	5-fold Cross Validation

## Discussion

Based on the results shown in Table 2 and Table 3, the accuracy for each fold and the average accuracy increases by increasing both the window size and the number of channels. Doubling the number of electrode channels from 32 to 64 results in $2-3$%, and from 64 to 128 in $1-2$% increase in all the reported accuracies. Intuitively speaking, on the one hand, increasing the window size feeds more data to the model at each epoch, which can enhance its performance as the difference among various gestures is more detectable through larger window sizes. On the other hand, instead of increasing the skip step while increasing the window size, we kept the skip step constant at 32 to feed more data to the model. In this scenario, the model has access to much more different samples of the training data as such possibly better learns the underlying representations of the data compared to the scenario where the skip step is larger but the model is fed with fewer data samples. Therefore, the model could be more generalizable while avoiding overfitting over to the train samples. Generalization refers to the ability of the model to make correct predictions for previously unseen data samples. More specifically, although the model is tested with completely unseen data samples, it has seen more samples during the training phase as such should be able to more effectively detect the underlying patterns among different gestures as such perform better on the unseen test data. The small skip step (32) chosen here means that the predictions are made every 15.3ms, causing a very small latency for real-time implementation of the proposed network in prosthetic devices. As it is evident from Table 2, starting from 86.23%, the average accuracy increases by $0.3-0.8$% each time the window size is increased reaching 91.98% when the window size and the number of channels are at the maximum. Therefore, the number of utilized channels, in general, has a greater impact on the accuracy in comparison to the window size. Moreover, the smallest accuracy is for *Fold*1 while the highest is for *Fold*3/*Fold*4, which could be due to the fact that in the first repetition, the subject was not completely aware of the procedure and how to exactly perform the required gesture. Intuitively speaking, the subject was being trained to perform the requested task. We hypothesize that, in the 3rd and 4th repetitions, the subject might have completely learned about the gesture and performed it more consistently, however, in the 5th repetition, fatigue might be a factor resulting in lower performance and relatively large drop in the accuracy.

**Figure 12:**
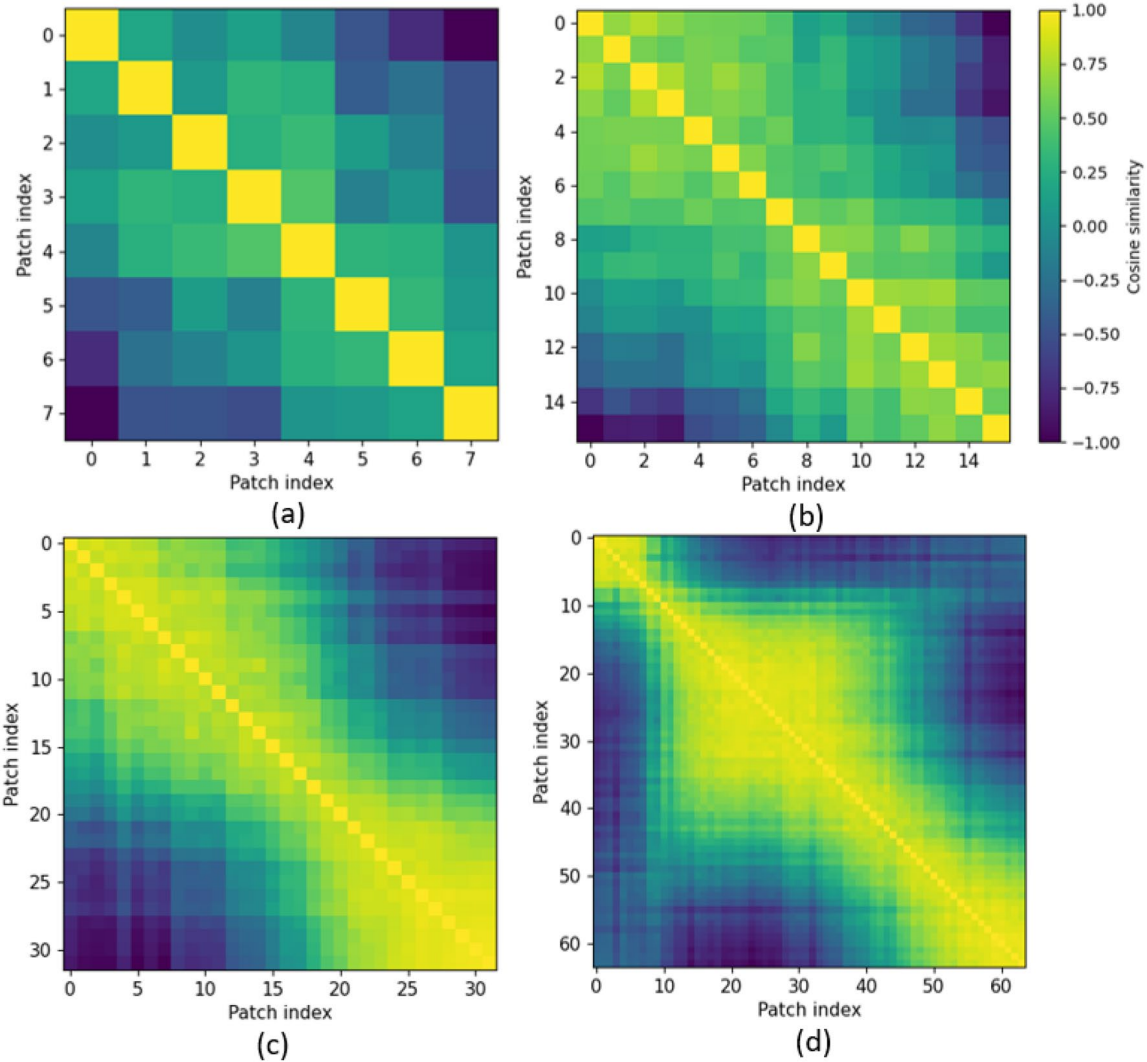
Cosine similarities of repetition 3, subject 20 of $\text {CT-HGR}$-V1 for (**a**) $W=64$ (**b**) $W=128$ (**c**) $W=256$ and (**d**) $W=512$.

**Figure 13:**
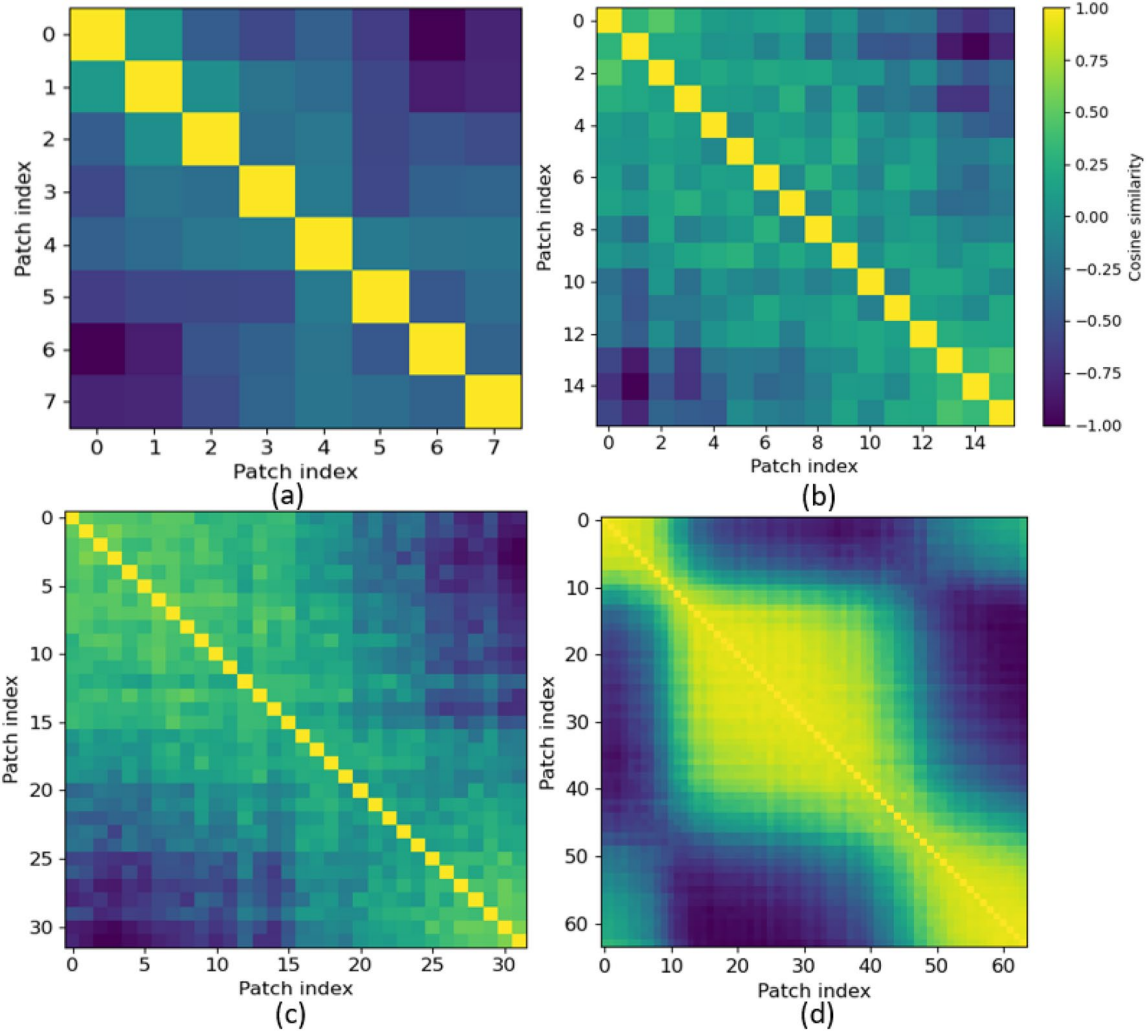
Cosine similarities of repetition 3, subject 20 of $\text {CT-HGR}$-V2 for (**a**) $W=64$ (**b**) $W=128$ (**c**) $W=256$ and (**d**) $W=512$.

As can be seen from Fig. 5, choosing the first repetition as the test set considerably differs from choosing the third or fourth repetition as the former yields much lower accuracy on average. STD for each fold and their average follows the same pattern as that of the accuracy, however, in an opposite direction, meaning that the best accuracy is usually associated with the least STD. This issue justifies the difference between the acquired accuracy in our proposed $\text {CT-HGR}$-V1 model with that of References^41,42,44^ using the same HD-sEMG dataset^31^. As mentioned before, two ML/DL models could be fairly comparable only if their train/test datasets are similar.

As can be seen in Table 3, Model $\text {CT-HGR}$-V2 is generally a better model compared to its $\text {CT-HGR}$-V1 variant as the accuracy for each fold and the overall average are higher. This is because $\text {CT-HGR}$-V2 is a bigger model with larger embedding dimension than $\text {CT-HGR}$-V1 in which the variations among different patches are more effectively embedded helping it to better discriminate between different hand gestures. Nevertheless, while the best improvement in accuracy occurs for *Fold*1 with $\approx$ 1.5% increase compared to $\text {CT-HGR}$-V1, not much improvement (less than 1% in most cases) is observed in the other folds and the final average. As indicated in Table 4, $\text {CT-HGR}$-V2’s number of learnable parameters is roughly 3 times the number of learnable parameters of $\text {CT-HGR}$-V1, however, there is a marginal progress in its performance in comparison to the former model. This shows that the hyperparameters used in $\text {CT-HGR}$-V1, producing no more than 100, 000 learnable parameters for the model, are sufficient for learning the 66 hand movements with high accuracy and there is no need to use more complex models for hand gesture classification using the proposed $\text {CT-HGR}$ framework on this specific HD-sEMG dataset. Clearly, deploying more complex models takes more memory and training time, which in turn reduces the overall efficiency of the model. According to the box plots shown in Fig. 6, all the comparisons between different window sizes are statistically significant. According to our results and those of^41^, in the case of HD-sEMG data, changing the window size has a great impact on the model’s accuracy in contrast to sparse sEMG signals. In HD-sEMG signals, thanks to using large number of electrode channels, there exists valuable information about differentiable patterns among hand gestures even in small window sizes. We should also mention that there exists a direct link between the window size and responsiveness in prosthetics^71^. For $\text {CT-HGR}$-V2, we have $p \le 0.001$ for the $W=64$ / $W=128$ and $W=256$ / $W=512$ pairs, which is less statistically significant than the other pairs with $p \le 0.0001$. For $\text {CT-HGR}$-V2, the results for the $W=64$ / $W=128$ pair are with $p \le 0.05$ which is less statistically significant than that for the other pairs. In our experiments, we aimed to verify that our proposed model can extract the underlying patterns in a single sample or very small portion of HD-sEMG data while these patterns are not easily discernible in sparse sEMG data. Although this may not be widely used in today’s real-time HMI devices, it can be a potential field of research and development of the current devices for window sizes of 2 ms and below to evaluate user’s experience.

As mentioned previously, the positional embedding used in the $\text {CT-HGR}$ framework is a 1D trainable embedding vector that is added to each of the embedded patches. By increasing the window size in our experiments, the patch size remains constant and the number of patches increases. This causes the positional embedding, which is the principal factor in determination of the input samples’ succession, to learn the positions more precisely. Fig. 12 illustrates the cosine similarity matrices of the positional embedding in Model $\text {CT-HGR}$-V1. Cosine similarities are sketched for different window sizes, 128 electrode channels and the trained model on subject 20 when repetition 3 is considered as the test set. In this case, models with window sizes of 64, 128, 256, and 512 have (8, 16) patch sizes. Therefore, each contain 8, 16, 32 and 64 patches in total. The *x* and *y* coordinates show the patch indices for each case and each row shows the similarities between each patch and the other patches. The diagonal values in each matrix are the largest values because their positional embedding vector is the same and its cosine is maximum. Similarity in the learned positional embedding vector of patches declines as the patches become farther. For $W=512$, the model learns the positions better and cosine similarities change more smoothly. Fig. 13 demonstrates the cosine similarity matrices of the positional embedding in Model $\text {CT-HGR}$-V2. Evidently, Model $\text {CT-HGR}$-V2 has learned the position embeddings more effectively as there is less similarity between the distant patches for all the window sizes. The more the window size increases, the more the model discriminates between the distant patches and the more the adjacent patches are considered similar to each other. As illustrated in Figs. 12 and  13, for $W=512$, Model $\text {CT-HGR}$-V2 behaves in a more orderly fashion than Model $\text {CT-HGR}$-V1 and consequently, extracts the positional information better.

Regarding instantaneous training, authors in^30^ implemented a CNN to conduct instantaneous classification of 8 gestures in the CapgMyo DB-a dataset. They applied various pre-processing and hyperparameter tuning steps and achieved the best performance of 89.3 for 18 subjects and 8 different gestures when all the 128 channels of the electrode grid were utilized. However, we achieved average accuracy of 89.13% for 19 subjects and 66 hand gestures with 64 channels. It is worth mentioning that 89.13 for 19 subjects and 66 gestures is achieved with the lightest version of our framework. Based on the results shown in Table 9, no significant discrepancy between the results for instantaneous training and larger window sizes is found. The results, in this case, are very similar to that of $\text {CT-HGR}$-V1, when W=128 and number of channels is equal to 64. This suggests that instantaneous training can sometimes work even better than training on very large window sizes with our proposed framework. More specifically, the model is able to achieve high accuracy in learning 66 hand movements with a single-point input which can be considered as an important breakthrough in the field of hand gesture recognition. This proves that HD-sEMG datasets provide highly valuable information of the muscles’ activity in each time point which are sufficient for the model to learn various hand gestures with no need for larger window sizes. Furthermore, training with single-point windows of data provides a great number of input samples to the $\text {CT-HGR}$ which helps the model generalize better and avoid overfitting. Based on the results shown in Table 8, the average accuracy and STD with shuffling is $\approx$ 9% higher and $\approx$ 1.4% lower than the results of the 5-fold cross-validation, respectively. This, however, can cause major issues in practice when dealing with hand prosthetic devices since the test data is entirely unseen and the pre-trained model could not perform reliably while testing with new datasets. In other words, the results reported without shuffling should be used as the bases for practical utilization.

Based on the results shown in Table 6 and Fig. 10, contrary to $\text {CT-HGR}$, increasing the window size leads to significant improvements in the average accuracy of the conventional ML models. In general, the achieved accuracy for the best performing ML models, i.e., SVM-V1 and LDA-V1 (trained with a newly proposed set of features), is $3-6$% lower and $0.5-0.8$% higher than $\text {CT-HGR}$-V1 with $W=64$ and $W=256$, respectively. Furthermore, as indicated in Table 6 and Table 4, our proposed $\text {CT-HGR}$-V1 framework surpasses the 3D CNN model by $\approx$ 3% average accuracy while employing less than 1/4 of the learnable parameters used in the 3D CNN model. According to Table 6 and Fig. 10, the accuracy of both the deep networks ($\text {CT-HGR}$-V1 and 3D CNN) increases by less than 1% with doubling the window size. As shown in Fig. 10, there is statistically significant difference among the six models with window size of 64 ($p \le 0.0001$), implying that the proposed $\text {CT-HGR}$-V1 gives its best performance at smaller window sizes. For $W=128$, the difference between $\text {CT-HGR}$-V1 and SVM-V1 and LDA-V2 is not significant although these models achieve twice the STD of $\text {CT-HGR}$-V1. The proposed $\text {CT-HGR}$-V1 model seems to perform similarly to SVM-V1, LDA-V1 and SVM-V2 models when the window size is set to 256 as the Fig. 10 shown no significant discrepancy in the average accuracy of these models. In this case, there is still significant difference between $\text {CT-HGR}$-V1 and 3D CNN architectures with $p \le 0.0001$.

According to Table 7, the train and test times for the two LDA models are less than that of $\text {CT-HGR}$-V1 while the maximum allocated memory for ML models with the second set of features that resulted in better accuracy is much higher than the maximum memory requirement of the $\text {CT-HGR}$-V1. This can be attributed to fact that the process of extracting five features from each channel of the HD-sEMG signals requires a great amount of system memory. On the contrary, DL-based models do not need a separate feature extraction step and the input windowed signals are the only item that needs system’s memory allocation. It is worth nothing that when it comes to the train time, $\text {CT-HGR}$-V1 needs 20 epochs to secure the minimum loss and the best convergence of the model. However, if the $\text {CT-HGR}$-V1 model is run with even 10 epochs, the accuracy drops around 0.8%, but the train time halves, i.e., 189 seconds. As stated previously, the train and test times are calculated in seconds for training the whole signal of one complete repetition for one subject. The batch size used for the testing stage of the $\text {CT-HGR}$-V1 is set equal to that of the training phase, i.e., 128. This impacts the test time of the $\text {CT-HGR}$-V1 (with larger batch sizes, the test time should reduce) compared to the ML models where the whole test data is provided at once. As can be seen in Table 7, the test time for the 3D CNN model is the least, but it has much larger training time, larger number of trainable parameters and less accuracy in comparison to $\text {CT-HGR}$-V1.

Based on Fig. 9, $\text {CT-HGR}$-V1 architecture performs poorly for gestures 57 and 59 as it achieves low precision, recall and F1 score for these two gestures. Gesture 36, also, in this model has a low recall measure implying that of all the samples that were labelled as class 36, not a great number of them were labelled correctly by $\text {CT-HGR}$-V1. SVM-V1 model was also incapable of effectively classifying gestures 57 and 59, but acted more precisely than $\text {CT-HGR}$-V1 for these gestures. This model, however, performs worse than $\text {CT-HGR}$-V1 on gesture 64 in terms of precision and F1 score. According to Table 10 in which the studies are reported for the 250 ms window size, $\text {CT-HGR}$-V1’s accuracy is higher than that of the $\text {CT-HGR}$-V3 by $\approx$ $3-4$ %, except *Fold*1 for which the peak-to-peak values of MUAPs provide more accurate information of the performed hand gesture than the HD-sEMG signals. However, a great improvement in average performance of the fused model in comparison to both stand-alone models is witnessed which is 8.22 and 5.52 % increase compared to $\text {CT-HGR}$-V1 and V3, respectively. As a side note on current challenges in EMG-based control of prosthetic hands, according to Reference^72^, one of the future perspectives to achieve the real-time usability of prosthetic, is to improve the feature extraction component of the EMG-based solutions. Deep learning is envisioned as one fruitful approach to address the feature extraction problem, which is the focus of this study. When it comes to real-time continuous classification, beside achieving high accuracies, one requires rapid response. The proposed framework provides high accuracies over small window sizes, therefore, can generate fast and dense decision flows. In summary, we hypothesized that by introducing a compact DL-based model that has the capacity to classify a large number of hand gestures with a small amount of memory and training time, we can put a step forward towards development of more dextrous control interfaces.

As a final remark, here we focus on clarifying specific questions related to the overall design of the proposed framework. The first question that comes to the mind is how to extract the MUAP in real-time. The decomposition method utilizing STA (from extracting MUSTs to obtaining MUAPs) is performed offline, which is considered as a limitation of the method as stated in the Sect. "Conclusion". Real-time extraction of MUAPs is a fruitful direction for future research and our suggested intuition is to design a DL-based model for extraction of MUSTs in real-time. Another question is on the rational of the statement that the MUAP in the sliding window contains information on MU recruitment. MUSTs show temporal activities of each MU in the course of performing different hand gestures. Duration of signals for each hand gesture in our dataset is about 4.5 seconds, therefore, during the entire process of performing a hand movement, different MUs with different levels of activities (forces) are involved. Consequently, extracting MUAPs based on small segments of the whole signal can provide us with more accurate information on MU recruitment at each stage of performing a specific hand gesture. Authors in References^73,74^ have also adopted a similar measure to perform STA by using sliding windows of various sizes based on their application. In^74^, it is explained that since the force level changes during performing a hand gesture, sliding STA is used to obtain detailed information of the MU recruitments within small time intervals. Another key question is the rational behind integration of MUAP with raw EMG signals. Intuitively speaking, each of these signals provide different information about how a specific hand gesture was performed. HD-sEMG signals reflect the macroscopic view of the neural drive information when performing a hand gesture. These signals provide useful information about amplitude variation, signal envelope, and onset/offset times of muscle contraction which are all extracted from the signals on the skin surface. However, MUAPs represent a microscopic view of the neural drive which is very similar to the behavior of human’s brain and individual motor neurons when a hand movement is being performed. This includes information about MU recruitments, MU firing rates, MU size/shape and MUAP amplitudes which are not readily provided by raw HD-sEMG signals. As the two signals are relevant to different parts of body and provide distinct views of macroscopic and microscopic neural drive information, we combined them to achieve more accurate classification accuracy for the gesture recognition task.

## Conclusion

In this study, we proposed a ViT-based architecture, referred to as the $\text {CT-HGR}$ framework, for hand gesture recognition from HD-sEMG signals. Efficacy of the proposed $\text {CT-HGR}$ framework is validated through extensive set of experiments with various numbers of electrode channels and window sizes. Moreover, the proposed model is evaluated on instantaneous data samples of the input data, achieving, more or less, a similar accuracy to scenarios with larger window sizes. This provides the context for real-time learning from HD-sEMG signals. Although increasing the number of learnable parameters of the $\text {CT-HGR}$ network leads to higher accuracy, the network works reasonably well on 66 hand gestures with less than 65k number of learnable parameters. This is exceptional as its conventional DL-based counterparts have, at times, millions of parameters. Besides, a hybrid model that is trained on raw HD-sEMG signals and their decomposed MUAPs is introduced, which substantially enhances the accuracy of the single $\text {CT-HGR}$ model trained solely on raw HD-sEMG data.

Although the utilized HD-sEMG dataset in this study is a comprehensive dataset acquired for a large number of hand gestures and from various subjects, it is obtained only from able-bodied individuals. This can be considered as a limitation of our developments. One direction for future works is to incorporate neurophysiological characteristics of hand amputees by acquiring a more generalized dataset that includes signals from this population. Moreover, the HD-sEMG decomposition phase in this study is conducted offline, preventing the proposed hybrid model to be employed in real-time HMI devices. This can be considered another limitation of our developments and a second fruitful direction for the future work to design a DL-based architecture for extracting MUSTs in real-time for development of online HMI systems. Another fruitful and important direction for future research is to focus on explainable AI to represent the extracted feature space through the proposed network and compare it with that of the conventional ML models. Finally, it would be interesting and intuitively pleasing to research potentials of Spiking Neural Networks (SNN) in this domain.

## Data Availability

The utilized dataset is publicly available through the following link: https://doi.org/10.6084/m9.figshare.c.5090861^31^.

## References

[1] Li W, Shi P, Yu H (2021). Gesture recognition using surface electromyography and deep learning for prostheses hand: State-of-the-art, challenges, and future. Front. Neurosci..

[2] Rahimian E (2021). Fs-hgr: Few-shot learning for hand gesture recognition via electromyography. IEEE Trans. Neural Syst. Rehabil. Eng..

[3] Rahimian, E. *et al.* Hand gesture recognition using temporal convolutions and attention mechanism. In *ICASSP 2022-2022 IEEE International Conference on Acoustics, Speech and Signal Processing (ICASSP)*, 1196–1200 (IEEE, 2022).

[4] Farina D, Mohammadi A, Adali T, Thakor NV, Plataniotis KN (2021). Signal processing for neurorehabilitation and assistive technologies. IEEE Signal Process. Mag..

[5] Tam S, Boukadoum M, Campeau-Lecours A, Gosselin B (2021). Intuitive real-time control strategy for high-density myoelectric hand prosthesis using deep and transfer learning. Sci. Rep..

[6] Chen, W. & Zhang, Z. Hand gesture recognition using semg signals based on support vector machine. In *2019 IEEE 8th Joint International Information Technology and Artificial Intelligence Conference (ITAIC)*, 230–234 (IEEE, 2019).

[7] Lee KH, Min JY, Byun S (2021). Electromyogram-based classification of hand and finger gestures using artificial neural networks. Sensors.

[8] Leone F (2019). Simultaneous semg classification of hand/wrist gestures and forces. Front. Neurorobot..

[9] Zhang R, Zhang X, He D, Wang R, Guo Y (2022). semg signals characterization and identification of hand movements by machine learning considering sex differences. Appl. Sci..

[10] Emayavaramban G (2021). Semg based classification of hand gestures using artificial neural network. Mater. Today Proc..

[11] Rahimian, E., Zabihi, S., Atashzar, S. F., Asif, A. & Mohammadi, A. Semg-based hand gesture recognition via dilated convolutional neural networks. In *2019 IEEE Global Conference on Signal and Information Processing (GlobalSIP)*, 1–5 (IEEE, 2019).

[12] Chen X, Li Y, Hu R, Zhang X, Chen X (2020). Hand gesture recognition based on surface electromyography using convolutional neural network with transfer learning method. IEEE J. Biomed. Health Inform..

[13] Azhiri, R. B., Esmaeili, M. & Nourani, M. Real-time emg signal classification via recurrent neural networks. In *2021 IEEE International Conference on Bioinformatics and Biomedicine (BIBM)*, 2628–2635 (IEEE, 2021).

[14] Simão M, Neto P, Gibaru O (2019). Emg-based online classification of gestures with recurrent neural networks. Pattern Recogn. Lett..

[15] Rahimian, E. *et al.* Temgnet: Deep transformer-based decoding of upperlimb semg for hand gestures recognition. *arXiv preprint* arXiv:2109.12379 (2021).

[16] Toledo-Peral, C. L. *et al.* semg signal acquisition strategy towards hand fes control. *J. Healthcare Eng.* **2018** (2018). 10.1155/2018/2350834 PMC5872608 29732046

[17] Jiang N, Dosen S, Muller K-R, Farina D (2012). Myoelectric control of artificial limbs-is there a need to change focus?[in the spotlight]. IEEE Signal Process. Mag..

[18] Kuruganti U, Pradhan A, Toner J (2021). High-density electromyography provides improved understanding of muscle function for those with amputation. Front. Med. Technol..

[19] Ketykó, I., Kovács, F. & Varga, K. Z. Domain adaptation for semg-based gesture recognition with recurrent neural networks. In *2019 International Joint Conference on Neural Networks (IJCNN)*, 1–7 (IEEE, 2019).

[20] Rojas-Martínez M, Mañanas MA, Alonso JF (2012). High-density surface emg maps from upper-arm and forearm muscles. J. Neuroeng. Rehabil..

[21] Bai, D., Chen, S. & Yang, J. Upper arm motion high-density semg recognition optimization based on spatial and time-frequency domain features. *J. Healthcare Eng.* **2019** (2019). 10.1155/2019/3958029 PMC6452563 31080576

[22] Chen J, Bi S, Zhang G, Cao G (2020). High-density surface emg-based gesture recognition using a 3d convolutional neural network. Sensors.

[23] Rojas-Martínez M (2020). High-density surface electromyography signals during isometric contractions of elbow muscles of healthy humans. Scientific data.

[24] Campanini, I., Disselhorst-Klug, C., Rymer, W. Z. & Merletti, R. Surface emg in clinical assessment and neurorehabilitation: Barriers limiting its use. *Front. Neurol.* 934 (2020). 10.3389/fneur.2020.00934 PMC7492208 32982942

[25] Yang K, Xu M, Yang X, Yang R, Chen Y (2021). A novel emg-based hand gesture recognition framework based on multivariate variational mode decomposition. Sensors.

[26] Hu Y (2018). A novel attention-based hybrid cnn-rnn architecture for semg-based gesture recognition. PLoS ONE.

[27] Xu P, Li F, Wang H (2022). A novel concatenate feature fusion rcnn architecture for semg-based hand gesture recognition. PLoS ONE.

[28] Shen S, Wang X, Mao F, Sun L, Gu M (2022). Movements classification through semg with convolutional vision transformer and stacking ensemble learning. IEEE Sens. J..

[29] Vaswani, A. *et al.* Attention is all you need. *Adv. Neural Inf. Process. Syst.* **30** (2017).

[30] Geng W (2016). Gesture recognition by instantaneous surface emg images. Sci. Rep..

[31] Malešević N (2021). A database of high-density surface electromyogram signals comprising 65 isometric hand gestures. Sci. Data.

[32] Lopes J (2017). Hand/arm gesture segmentation by motion using imu and emg sensing. Procedia Manuf..

[33] Zhang Y, Yang F, Fan Q, Yang A, Li X (2022). Research on semg-based gesture recognition by dual-view deep learning. IEEE Access.

[34] Atzori M, Cognolato M, Müller H (2016). Deep learning with convolutional neural networks applied to electromyography data: A resource for the classification of movements for prosthetic hands. Front. Neurorobot..

[35] Wei W (2019). A multi-stream convolutional neural network for semg-based gesture recognition in muscle-computer interface. Pattern Recogn. Lett..

[36] Atzori M (2014). Electromyography data for non-invasive naturally-controlled robotic hand prostheses. Sci. Data.

[37] Koiva, R., Hilsenbeck, B. & Castellini, C. Evaluating subsampling strategies for semg-based prediction of voluntary muscle contractions. In *2013 IEEE 13th International Conference on Rehabilitation Robotics (ICORR)*, 1–7 (IEEE, 2013).

[38] Lopes J (2017). Hand/arm gesture segmentation by motion using imu and emg sensing. Procedia Manufacturing.

[39] Zhang Z, Yang K, Qian J, Zhang L (2019). Real-time surface emg pattern recognition for hand gestures based on an artificial neural network. Sensors.

[40] Rahimian, E., Zabihi, S., Atashzar, S. F., Asif, A. & Mohammadi, A. Xceptiontime: independent time-window xceptiontime architecture for hand gesture classification. In *ICASSP 2020-2020 IEEE International Conference on Acoustics, Speech and Signal Processing (ICASSP)*, 1304–1308 (IEEE, 2020).

[41] Khushaba RN, Nazarpour K (2021). Decoding hd-emg signals for myoelectric control-how small can the analysis window size be?. IEEE Robot. Autom. Lett..

[42] Massa, S. M., Riboni, D. & Nazarpour, K. Graph neural networks for hd emg-based movement intention recognition: An initial investigation. In *2022 IEEE International Conference on Recent Advances in Systems Science and Engineering (RASSE)*, 1–4 (IEEE, 2022).

[43] Sun, T., Libby, J., Rizzo, J. & Atashzar, S. F. Deep augmentation for electrode shift compensation in transient high-density semg: Towards application in neurorobotics. In *2022 IEEE/RSJ International Conference on Intelligent Robots and Systems (IROS)*, 6148–6153 (IEEE, 2022).

[44] Al Taee, A. A., Khushaba, R. N., Zia, T. & Al-Jumaily, A. The effectiveness of narrowing the window size for ld & hd emg channels based on novel deep learning wavelet scattering transform feature extraction approach. In *2022 44th Annual International Conference of the IEEE Engineering in Medicine & Biology Society (EMBC)*, 3698–3701 (IEEE, 2022). 10.1109/EMBC48229.2022.9871473 36086593

[45] Barsakcioglu, D. Y. & Farina, D. A real-time surface emg decomposition system for non-invasive human-machine interfaces. In *2018 IEEE Biomedical Circuits and Systems Conference (BioCAS)*, 1–4 (IEEE, 2018).

[46] Devlin, J., Chang, M.-W., Lee, K. & Toutanova, K. Bert: Pre-training of deep bidirectional transformers for language understanding. *arXiv preprint* arXiv:1810.04805 (2018).

[47] Ba, J. L., Kiros, J. R. & Hinton, G. E. Layer normalization. *arXiv preprint* arXiv:1607.06450 (2016).

[48] Dosovitskiy, A. *et al.* An image is worth 16x16 words: Transformers for image recognition at scale. *arXiv preprint* arXiv:2010.11929 (2020).

[49] Côté-Allard U (2019). Deep learning for electromyographic hand gesture signal classification using transfer learning. IEEE Trans. Neural Syst. Rehabil. Eng..

[50] Lee KH, Min JY, Byun S (2021). Electromyogram-based classification of hand and finger gestures using artificial neural networks. Sensors.

[51] Chen, H., Tong, R., Chen, M., Fang, Y. & Liu, H. A hybrid cnn-svm classifier for hand gesture recognition with surface emg signals. In *2018 International Conference on Machine Learning and Cybernetics (ICMLC)*, vol. 2, 619–624 (IEEE, 2018).

[52] Islam MJ (2021). A novel signal normalization approach to improve the force invariant myoelectric pattern recognition of transradial amputees. IEEE Access.

[53] Shen C (2022). Toward generalization of semg-based pattern recognition: A novel feature extraction for gesture recognition. IEEE Trans. Instrum. Meas..

[54] Khushaba RN, Al-Timemy AH, Al-Ani A, Al-Jumaily A (2017). A framework of temporal-spatial descriptors-based feature extraction for improved myoelectric pattern recognition. IEEE Trans. Neural Syst. Rehabil. Eng..

[55] Islam MJ (2022). Application of min-max normalization on subject-invariant emg pattern recognition. IEEE Trans. Instrum. Meas..

[56] Asogbon MG (2020). Towards resolving the co-existing impacts of multiple dynamic factors on the performance of emg-pattern recognition based prostheses. Comput. Methods Programs Biomed..

[57] Molchanov, P., Gupta, S., Kim, K. & Kautz, J. Hand gesture recognition with 3d convolutional neural networks. In *Proceedings of the IEEE conference on computer vision and pattern recognition workshops*, 1–7 (2015).

[58] Chen J, Bi S, Zhang G, Cao G (2020). High-density surface emg-based gesture recognition using a 3d convolutional neural network. Sensors.

[59] Alnuaim, A. *et al.* Human-computer interaction with hand gesture recognition using resnet and mobilenet. *Comput. Intell. Neurosci.* **2022** (2022). 10.1155/2022/8777355 PMC8976610 35378817

[60] Negro F, Muceli S, Castronovo AM, Holobar A, Farina D (2016). Multi-channel intramuscular and surface emg decomposition by convolutive blind source separation. J. Neural Eng..

[61] Chabriel G (2014). Joint matrices decompositions and blind source separation: A survey of methods, identification, and applications. IEEE Signal Process. Mag..

[62] de Oliveira DS (2022). Neural decoding from surface high-density emg signals: influence of anatomy and synchronization on the number of identified motor units. J. Neural Eng..

[63] Clarke AK (2020). Deep learning for robust decomposition of high-density surface emg signals. IEEE Trans. Biomed. Eng..

[64] Farina D (2017). Man/machine interface based on the discharge timings of spinal motor neurons after targeted muscle reinnervation. Nature Biomed. Eng..

[65] Holobar, A. & Zazula, D. Gradient convolution kernel compensation applied to surface electromyograms. In *International Conference on Independent Component Analysis and Signal Separation*, 617–624 (Springer, 2007).

[66] Holobar A, Zazula D (2007). Multichannel blind source separation using convolution kernel compensation. IEEE Trans. Signal Process..

[67] Chen M, Zhou P (2015). A novel framework based on fastica for high density surface emg decomposition. IEEE Trans. Neural Syst. Rehabil. Eng..

[68] Zhao Y (2022). Decoding finger movement patterns from microscopic neural drive information based on deep learning. Med. Eng. Phys..

[69] Chen C (2020). Hand gesture recognition based on motor unit spike trains decoded from high-density electromyography. Biomed. Signal Process. Control.

[70] Kapelner T (2019). Predicting wrist kinematics from motor unit discharge timings for the control of active prostheses. J. Neuroeng. Rehabil..

[71] Farrell TR, Weir RF (2007). The optimal controller delay for myoelectric prostheses. IEEE Trans. Neural Syst. Rehabil. Eng..

[72] Parajuli N (2019). Real-time emg based pattern recognition control for hand prostheses: A review on existing methods, challenges and future implementation. Sensors.

[73] Hu X, William ZR, Nina LS (2013). Motor unit pool organization examined via spike-triggered averaging of the surface electromyogram. J. Neurophysiol..

[74] Del Vecchio A, Francesco N, Francesco F, Dario F (2017). Associations between motor unit action potential parameters and surface emg features. J. Appl. Physiol..

